# Uncoupling therapeutic from immunotherapy-related adverse effects for safer and effective anti-CTLA-4 antibodies in *CTLA4* humanized mice

**DOI:** 10.1038/s41422-018-0012-z

**Published:** 2018-02-20

**Authors:** Xuexiang Du, Mingyue Liu, Juanjuan Su, Peng Zhang, Fei Tang, Peiying Ye, Martin Devenport, Xu Wang, Yan Zhang, Yang Liu, Pan Zheng

**Affiliations:** 1 Center for Cancer and Immunology Research, Children’s Research Institute, Children’s National Health System, Washington, DC 20010 USA; 2 OncoImmune, Inc., Rockville, MD 20852 USA

## Abstract

Anti-CTLA-4 monoclonal antibodies (mAbs) confer a cancer immunotherapeutic effect (CITE) but cause severe immunotherapy-related adverse events (irAE). Targeting CTLA-4 has shown remarkable long-term benefit and thus remains a valuable tool for cancer immunotherapy if the irAE can be brought under control. An animal model, which recapitulates clinical irAE and CITE, would be valuable for developing safer CTLA-4-targeting reagents. Here, we report such a model using mice harboring the humanized *Ctla4* gene. In this model, the clinically used drug, Ipilimumab, induced severe irAE especially when combined with an anti-PD-1 antibody; whereas another mAb, L3D10, induced comparable CITE with very mild irAE under the same conditions. The irAE corresponded to systemic T cell activation and resulted in reduced ratios of regulatory to effector T cells (Treg/Teff) among autoreactive T cells. Using mice that were either homozygous or heterozygous for the human allele, we found that the irAE required bi-allelic engagement, while CITE only required monoallelic engagement. As with the immunological distinction for monoallelic vs bi-allelic engagement, we found that bi-allelic engagement of the *Ctla*4 gene was necessary for preventing conversion of autoreactive T cells into Treg cells. Humanization of L3D10, which led to loss of blocking activity, further increased safety without affecting the therapeutic effect. Taken together, our data demonstrate that complete CTLA-4 occupation, systemic T cell activation and preferential expansion of self-reactive T cells are dispensable for tumor rejection but correlate with irAE, while blocking B7-CTLA-4 interaction impacts neither safety nor efficacy of anti-CTLA-4 antibodies. These data provide important insights for the clinical development of safer and potentially more effective CTLA-4-targeting immunotherapy.

## Introduction

Anti-CTLA-4 monoclonal antibodies (mAbs) have shown a cancer immunotherapeutic effect (CITE) in mouse models^1–3^ and melanoma patients.^4,5^ In combination, the anti-PD-1 mAb, Nivolumab, and the anti-CTLA-4 mAb, Ipilimumab, significantly increased objective response rates in advanced melanoma patients.^6,7^ Promising results also emerged from this combination therapy in advanced non-small cell lung carcinoma (NSCLC).^8^ Similar clinical benefits were observed when another anti-CTLA-4 mAb (Tremelimumab) was combined with durvalumab, an anti-PD-L1 mAb.^9^ A major obstacle to the broader clinical use of anti-CTLA-4 mAbs, either alone or in combination, is the severe adverse events (SAEs).^6,7^ The SAEs observed in trials of Ipilimumab (the first clinical anti-CTLA-4 mAb) led to the concept of immunotherapy-related adverse events (irAE).^10^ In particular, combination therapy with Ipilimumab and Nivolumab (anti-PD-1) led more than 50% patients to develop grade 3 and grade 4 SAE. In NSCLC, Ipilimumab and Nivolumab combination therapy resulted in high response rates, although the grade 3 and 4 SAEs also occurred at high rates.^8^ Similarly, the combination of Durvalumab (anti-PD-L1) and Tremelimumab (another anti-CTLA-4 mAb in clinical testing) showed clinical activities in NSCLC, although grade 3 and 4 SAEs and patient drop-off rate were high, presumably due to unacceptable toxicity.^9^ Since a higher dose of anti-CTLA-4 mAb is associated with better clinical outcomes in both monotherapy and combination therapy, irAE not only prevents many patients from continuing immunotherapy, but also limits the efficacy of CITE. Furthermore, high numbers of patients drop-off trials with both anti-CTLA-4 mAbs, a finding likely attributable to failure to meet clinical endpoints.^11,12^

More recently, a head-to-head comparison of the anti-PD-1 mAb, Nivolumab, and the anti-CTLA-4 mAb, Ipilimumab, as adjuvant therapy for resected stage III and IV melanoma showed that Ipilimumab had a lower CITE but higher irAE,^13^ further dimming the prospect of CTLA-4-targeting immunotherapy. However, Ipilimumab-treated patients who survived for 3 years showed no further decline in survival rate over a 10-year period.^14^ This remarkably sustained response highlights the exceptional benefit of targeting this molecule for immunotherapy, especially if irAE can be brought under control.

A fundamental question for the generation of safe and effective anti-CTLA-4 mAbs is whether CITE and irAE are intrinsically linked. The classical checkpoint blockade hypothesis stipulates that anti-CTLA-4 mAbs promote cancer immunity by blocking a negative signal of the B7-CTLA-4 interaction to promote naïve T cell activation in the lymphoid organ.^15^ According to this model, therapeutic antibodies are antagonists that functionally inactivate CTLA-4:B7 interactions. Since genetic inactivation of CTLA-4 expression leads to autoimmune diseases in mouse and human, it is assumed that the irAE would be a necessary price to pay for CITE. On the other hand, recent studies suggest that rather than blocking the B7-CTLA-4 interaction, the therapeutic effect of anti-mouse CTLA-4 mAbs requires antibody-mediated depletion of regulatory T cells (Tregs) specifically within tumor microenvironment.^16–18^ These studies raise the intriguing possibility that CITE can be achieved without irAE if one can achieve local Treg cell depletion without mimicking genetic inactivation of CTLA-4 expression. In order to test this hypothesis, it is essential to establish a model that faithfully recapitulates clinically observed irAE.

Commonly reported irAE in patients receiving either anti-CTLA-4 or anti-CTLA-4 plus anti-PD-1/PD-L1 agents includes hematological abnormalities such as pure red cell aplasia,^19,20^ and non-infection-related inflammatory damage to solid organs, such as colitis, dermatitis, pneumonitis, hepatitis and myocarditis.^21–23^ While the term irAE implies an intrinsic link between CITE and autoimmune AE, there are very few investigational studies that substantiate such a link. In contrast, our previous study involving human *Ctla4* knock-in mice showed that the levels of anti-DNA antibodies and cancer rejection parameters do not always correlate with each other.^24^ In particular, we found that one of the antibodies tested, L3D10, conferred strongest CITE but yet induced the lowest levels of anti-DNA antibodies among several mAbs tested. Nevertheless, since the anti-CTLA-4 mAb-induced adverse events are relatively mild in mice, this model failed to recapitulate clinical observations. As such it is of limited value in understanding the pathogenesis of irAE and in identification of safe and effective anti-CTLA-4 mAbs. Moreover, since these studies were performed before clinically used anti-CTLA-4 mAbs were available, it is unclear, whether the principles are relevant to irAE induced by clinical products.

In developing a mouse model of irAE, we considered three factors. First, since combination therapy with anti-PD-1 and anti-CTLA-4 is being rapidly expanded into multiple indications, a model that recapitulates the combination therapy would be of great significance for the field. Second, the fact that combination therapy results in SAEs (grades 3 and 4 organ toxicity) in more than 50% of the subjects will make it easier to recapitulate irAE in the mouse model. Third, since the mouse is generally more resistant to irAE, one must search for conditions under which the irAE can be faithfully recapitulated. As the autoimmune phenotype in *Ctla4*^*−/−*^ mice occurs at a young age,^25,26^ and targeted mutation of the *Ctla4* gene in adult mice leads to less severe autoimmune disease,^27^ we reasoned that mice may be most susceptible to anti-CTLA-4 mAbs if they are administrated at a young age. Taking these factors into consideration, we now report a model system that faithfully recapitulates the irAEs observed in clinical trials of combination therapy. More importantly, by using different genetic models and therapeutic anti-CTLA-4 mAbs, we show that irAE and CITE are not intrinsically linked and they have a distinct genetic and immunological basis, as complete CTLA-4 occupation, systemic T cell activation and preferential expansion of self-reactive T cells are dispensable for tumor rejection but correlate with irAE. Moreover, blocking the B7-CTLA-4 interaction impacts neither safety nor efficacy of anti-CTLA-4 antibodies. Rather, our companion paper demonstrated that FcR-mediated Treg depletion in the tumor microenvironment is necessary and sufficient for tumor rejection. These results provide important insights for the therapeutic development of the next generation of safer and more effective anti-CTLA-4 antibodies.

## Results

### Human CTLA4 knock-in mice model faithfully recapitulates irAE of combination therapy

**Fig. 1:**
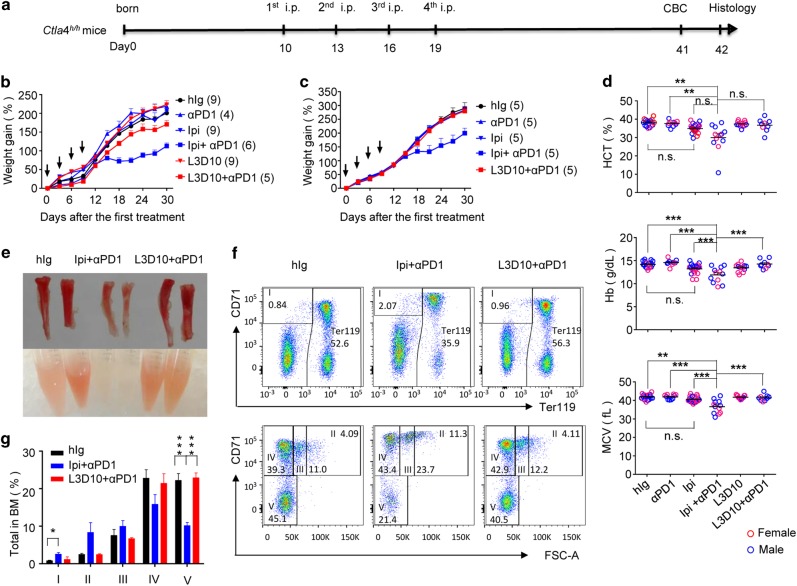
Human *CTLA4* gene knock-in mice distinguished irAE of anti-CTLA-4 mAbs Ipilimumab and L3D10 when used alone or in combination with anti-PD-1 mAb: growth retardation and pure red blood cell aplasia. **a** Timeline of antibody treatment and analysis. C57BL/6 *Ctla4*^*h/h*^ mice were treated, respectively, with control human IgG-Fc, anti-human CTLA-4 mAb Ipilimumab, human IgG1 Fc chimeric L3D10 + human IgG-Fc, anti-PD-1 (RMP1-14) + human IgG-Fc, anti-PD-1 + Ipilimumab or anti-PD-1 + L3D10 at a dose of 100 μg/mouse/injection on days 10, 13, 16 and 19. The CBC analysis was performed on day 41 after birth and necropsy was performed on day 42 after birth. To avoid cage variation, mice in the same cages were individually tagged and treated with different antibodies. Tests were performed double blind. **b** Major growth retardation of female mice by Ipilimumab + anti-PD-1. One female mouse from Ipilimumab plus anti-PD-1-treated group was excluded from analysis due to death on day 22 with serious grow retardation. Data shown were means and S.E.M. of % weight gain following the first injection. hIg vs Ipilimumab + anti-PD-1, *P* *<* 0.0001; L3D10 + anti-PD-1 vs Ipilimumab + anti-PD-1, *P* = 0.003. **c** Major growth retardation of male mice by Ipilimumab + anti-PD-1. As in **b**, except male mice were used. hIg vs Ipilimumab + anti-PD-1, *P* = 0.0116; L3D10 + anti-PD-1 vs Ipilimumab + anti-PD-1, *P* = 0.0152. The numbers of mice used were included in the parentheses following group labels. **d**–**g** Pure red cell aplasia recapitulated in the mouse model as a typical phenotype of irAE. **d** Ipilimumab + anti-PD-1 combination therapy reduced HCT, Hb and MCV. Data shown are a summary of 8 independent experiments with each dot representing one individual mouse (blue for male mice and red for female mice, and *n* = 9–22 mice per group. **e** Defective generation of red cells in bone marrow. Photographs depict the change of coloration in bone (upper panel) and bone marrow flush (lower panel) in mice that received indicated treatments. **f** Analysis of erythrocyte development by flow cytometry. Data shown are representative FACS profiles depicting distribution of Ter119, CD71 and forward scatters (FSC-A) among bone marrow cells. The gating and % of cells at stage I–V are indicated. **g** Summary data of % of erythroid cells at each of the developmental stages. Data shown are means and S.E.M. of data with 3–4 female mice per group, and have been repeated at least three times in both male and female mice. Statistical tests used: **b** and **c**, two-way repeat measurement ANOVA with Bonferroni multiple comparison test; **d** and **g**, one-way ANOVA with Bonferroni multiple comparison test and non-parametric one-way ANOVA (Kruskal–Wallis test) with Dunn’s multiple comparisons test

A major challenge in studying the mechanisms and preventive strategies of irAE in combination therapy is that the mouse tolerates high doses of anti-CTLA-4 mAb without significant AE. We choose two human CTLA-4 mAbs for this study: the clinically used Ipilimumab and L3D10, the most potent among our panel of anti-CTLA-4 mAbs.^24,28^ When compared in the same model, the two mAbs were comparable in causing tumor rejection (Supplementary information, Figure S1). Since young mice expressed higher levels of CTLA-4, recapitulating a feature of adult tumor-bearing mice (Supplementary information, Figure S2), we treated perinatal human *CTLA4* knock-in (*Ctla4*^*h/h*^) mice with control human IgG-Fc, anti-CTLA-4 mAb Ipilimumab, L3D10, anti-PD-1, anti-PD-1 + Ipilimumab or anti-PD-1 + L3D10, respectively. The mice were treated on days 10, 13, 16 and 19 after birth, at the doses of 100 μg/mouse/injection, and were evaluated for the rate of body weight gain over time, and for hematologic and histopathology alterations at 6 weeks of age (Fig. 1a). We found that whereas a combination of Ipilimumab and anti-PD-1 significantly retarded growth in female mice, either antibody alone did not have a major impact on body weight gain (Fig. 1b). Male mice also showed substantial and statistically significant growth retardation in response to anti-PD-1 + Ipilimumab (Fig. 1c). Remarkably, no growth retardation was observed when anti-PD-1 + L3D10 was used (Fig. 1b, c). To study the impact of combination therapy on hematopoiesis, we carried out total blood cell counts at 1 month after initiation of combination therapy (Supplementary information, Figure S3). We observed a significant reduction of blood hematocrit (HCT), total hemoglobin (Hb) and mean corpuscular volume (MCV) among the majority of the mice treated with Ipilimumab + anti-PD-1, while those that received L3D10 + anti-PD-1 were unaffected (Fig. 1d). The presentation of leukocytes was largely normal (Supplementary information, Figure S3). These data demonstrate that the combination of anti-PD-1 and Ipilimumab, but not that of anti-PD-1 and L3D10, causes anemia. As a single agent, Ipilimumab, but not L3D10, induced anemia in high proportion of young mice although the average reduction was not statistically significant. After necropsy, it is clear that red cell generation in the bone marrow was severely limited as the bones and the bone marrow flushed out from Ipilimumab + anti-PD-1-treated mice appeared pale whereas those of L3D10 + anti-PD-1-treated mice were comparable to ones from control IgG-treated mice (Fig. 1e). To quantitate the defects in the red cell lineage in the bone marrow, we analyzed the distribution of CD71 and Ter119 markers among the bone marrow cells as well as the cell sizes. These markers have been used to mark five stages of erythrocyte development: stage I, CD71^+^Ter119^−^; stage II, FSC-A^hi^CD71^+^Ter119^+^; stage III, FSC-A^mi^CD71^+^Ter119^+^; stage IV, FSC-A^lo^CD71^+^Ter119^+^; and stage V, CD71^−^Ter119^+^. As shown in Fig. 1f,g, anti-PD-1 + Ipilimumab-treated mice showed a significant increase of progenitor cells (stage I) and a reduction in the frequency of mature red blood cells (stage V) explaining their severe anemia. In contrast, L3D10- and anti-PD-1-treated mice exhibited a normal distribution and maturation of erythrocytes in the bone marrow.

**Fig. 2:**
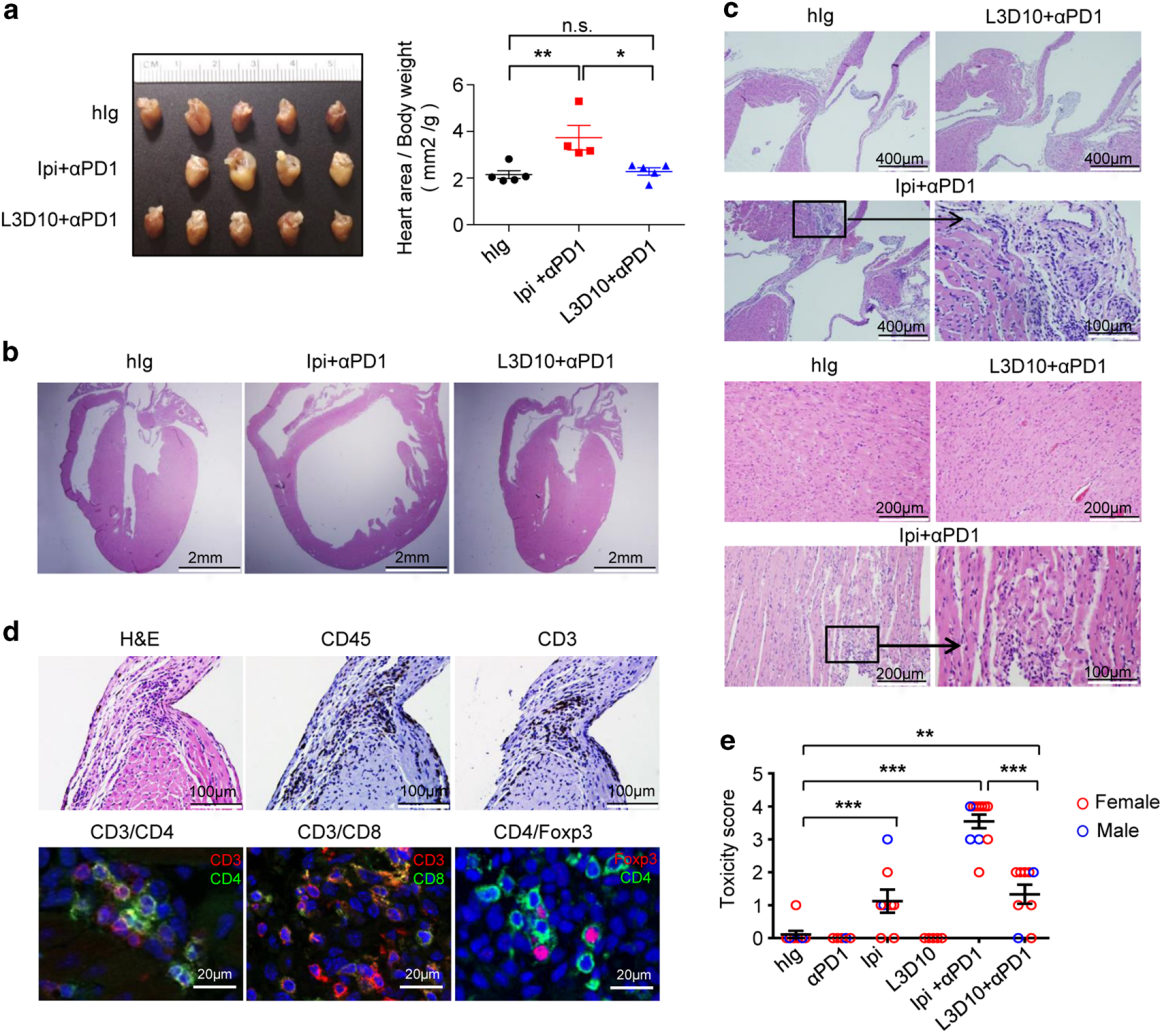
Ipilimumab caused heart defects when used in combination with anti-mouse PD-1. **a** Gross anatomy shows heart enlargement despite reduced body size in mice treated with anti-PD-1 + Ipilimumab. Photographs in the left panels are from formalin-fixed hearts from mice that received indicated treatments, and the data on the right panel show the sizes after normalizing against body weight. **b** Macroscopic images depicting enlarged heart atriums and ventricles, and corresponding thinning of heart wall. **c** Histology of control hIg, L3D10 + anti-PD-1 or anti-PD-1 + Ipilimumab-treated hearts. The upper four panels show H&E staining at the aorta base, while the lower four panels show inflammation in myocardium of the left ventricle. **d** Identification of leukocytes and T cells by immunohistochemistry (top panels) and three-color immunofluorescence staining using FITC-labeled CD4 or CD8, Rhodamine-labeled anti-CD3 or anti-Foxp3 antibodies (lower panel). **e** The composite pathology scores of male and female mice (*n* = 5–12) receiving different treatments. The scores of male mice are indicated with blue circles, while those of female mice are indicated with red circles. The samples were collected from six independent experiments and have been scored double blind. Data are mean ± S.E.M. and analyzed by one-way ANOVA with Bonferroni’s multiple comparison test

The dramatic difference in growth rate of *CTLA4*^*h/h*^ mice that received anti-PD-1 in conjunction with L3D10 vs Ipilimumab suggests that the two anti-CTLA-4 mAbs may induce very different AEs. To test this possibility, we sacrificed mice and performed necropsy when they reached 42 days of age. We observed marked cardiomegaly in anti-PD-1 + Ipilimumab-treated, but not in anti-PD-1 + L3D10-treated mice (Fig. 2a). The enlarged heart showed dilation of chambers of both the right and left ventricles, albeit more conspicuously in the left ventricle, indicating severe dilated cardiomyopathy. The left ventricular and ventricular sepal myocardium wall thickness decreased more than 50% in comparison with heart from the hIgG-treated group (Fig. 2b). The histology demonstrated myocarditis with diffuse and massive lymphocyte infiltrations in the endocardium and myocardium, degeneration of cardiomyocytes and structural disruptions at the inflammatory foci (Fig. 2c). High abundance of CD45^+^ and CD3^+^ T cells were observed in the hearts from anti-PD-1 + Ipilimumab-treated mice by immunohistochemistry (Fig. 2d, upper panels), consistent with a T cell-mediated pathology. These cells included both CD4 and CD8 T subsets (Fig. 2d, bottom panels). Foxp3^+^ CD4^+^ Treg cells were present at the inflammatory sites of anti-PD-1 + Ipilimumab-treated mice, suggesting that tissue destruction occurred despite the presence of Treg (Fig. 2d). Mild to moderate inflammation was observed in mice that received either L3D10 + anti-PD-1 combination therapy or Ipilimumab monotherapy. However, neither L3D10 nor anti-PD-1 monotherapy caused detectable inflammation (Fig. 2e). The fact that anti-PD-1 treatment failed to induce inflammation in heart may be attributed to the use of mice with the C57BL/6 background, which failed to develop heart diseases even when the *Pd1* gene was deleted,^29^ in contrast to mice with the BALB/c background. Apart from heart abnormalities, the combination of Ipilimumab and anti-PD-1 mAb also induced a severe defect in the female urinary-reproductive organs with histological findings of hypoplastic ovaries and uterus (Supplementary information, Figure S4). Consistent with defective adrenal gland function, we observed a significant elevation of adrenocorticotropic hormone, a likely response to defective production of cortisol by the adrenal gland (Supplementary information, Figure S5).

**Fig. 3:**
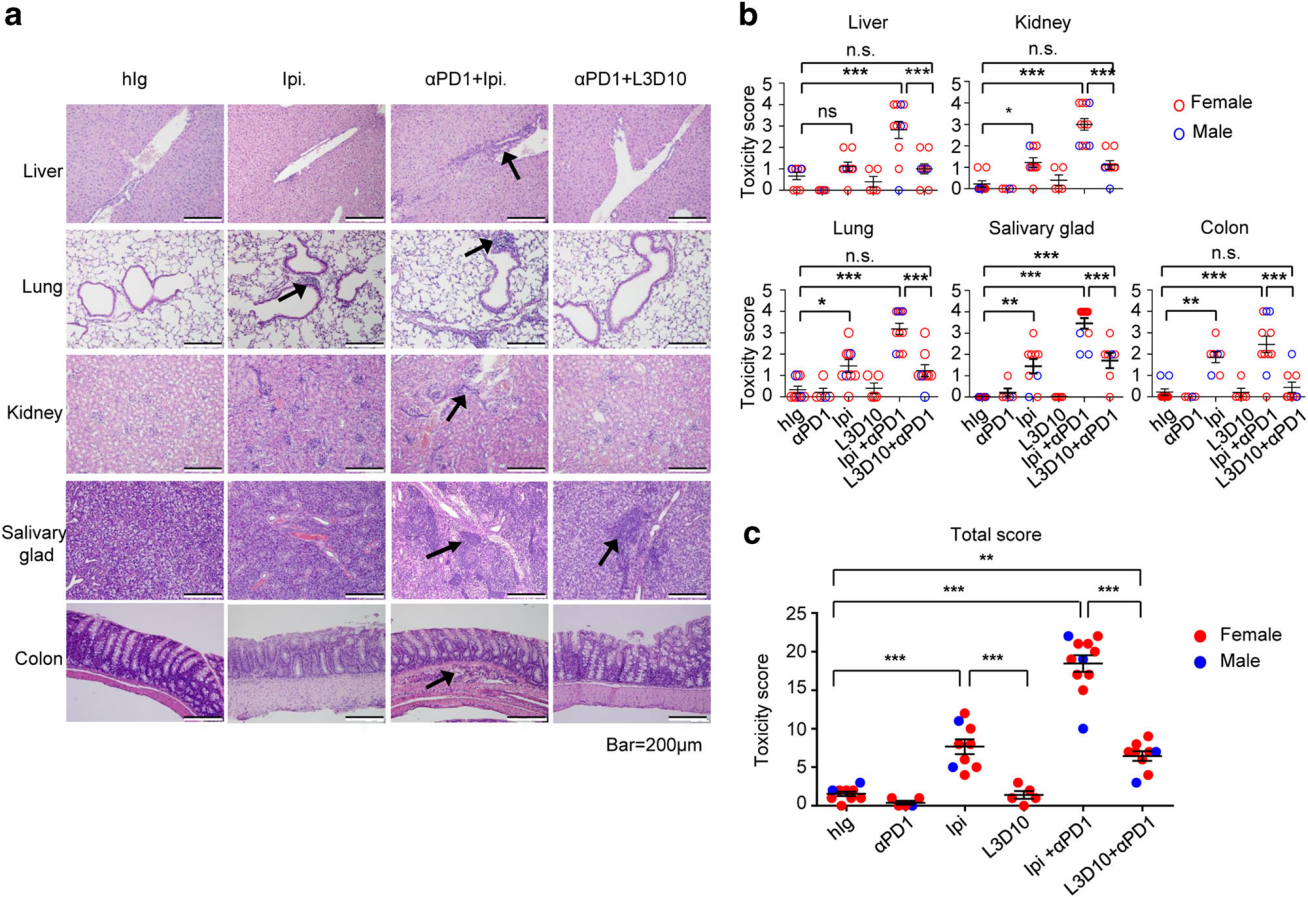
Ipilimumab caused multiple organ inflammation when either used as single agent or in combination with anti-PD-1. **a** Representative images of H&E stained paraffin sections from different organs. Representative inflammatory foci are marked with arrows. Scale bar, 200 μm. **b** Toxicity scores of internal organs and glands. The scores of male mice are indicated with blue circles, while that of female mice are indicated with red circles. **c** Composite scores of all organs and glands. Data are mean ± S.E.M., *n* = 5–12 mice per group. The samples were collected from six independent experiments and have been scored double blind. Data were analyzed by one-way ANOVA with Bonferroni’s multiple comparison test

To quantitatively analyze the impact of anti-PD-1, anti-CTLA-4 and their combinations on tissue destruction, we performed histological analysis of internal organs and glands from mice receiving either control Ig or immunotherapeutic antibodies. Organs and glands were fixed in 10% formalin, sectioned and stained with hematoxylin and eosin (H&E), and scored double blindly. Representative tissue sections are shown in Fig. 3a; scores from individual mice in each group are presented in Fig. 3b and composite scores of all organs are presented in Fig. 3c. Confirming its safety, L3D10 monotherapy failed to induce severe inflammation in any of the organs examined. In contrast, moderate to severe inflammation was induced by Ipilimumab monotherapy in all mice, which was significantly stronger than the occasional background inflammation in the control Ig, anti-PD-1 and L3D10 monotherapy groups. When combined with anti-PD-1, Ipilimumab-induced inflammation in all mice, and severe inflammation was found in all major organs. It is particularly noteworthy that transmural inflammation, which is the most severe form of histological findings in the colon and a unique pathological feature of Crohn’s disease, was observed in the anti-PD-1 and Ipilimumab-treated mice but was absent in other groups. When the scores from all organs were combined, it is clear that Ipilimumab + anti-PD-1-induced dramatically stronger inflammation than L3D10 + anti-PD-1 treatment (Fig. 3c). In addition, Ipilimumab alone also induced significantly stronger adverse events than either anti-PD-1 alone or L3D10 alone as single agents (Fig. 3c).

### Ipilimumab + anti-PD-1 but not L3D10 + anti-PD-1 induces systemic T cell activation and expansion of autoreactive effector T cells

**Fig. 4:**
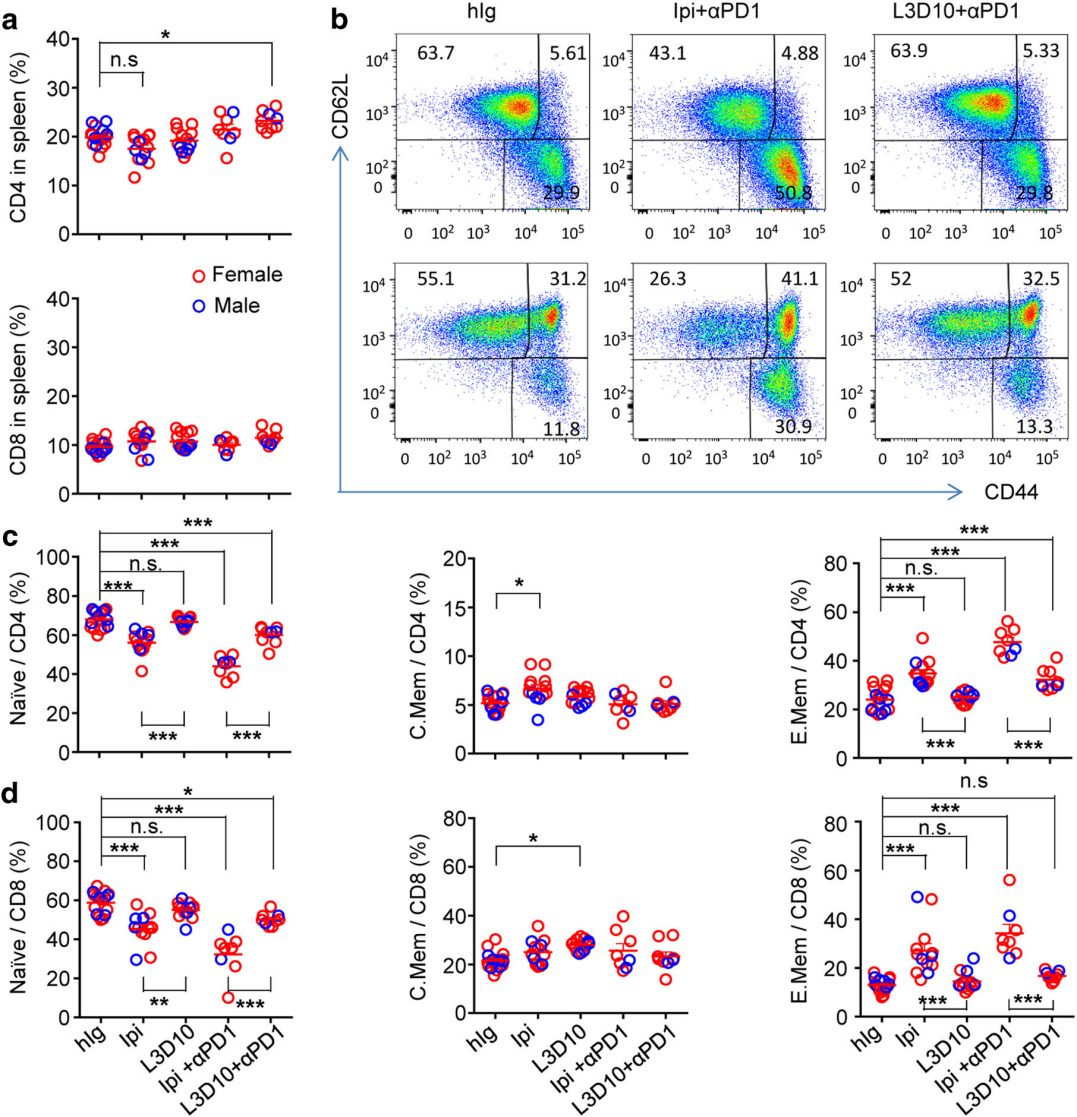
Comparison of systemic T cell activation in mice that received immunotherapy drugs starting at day 10. **a** Minor impact of CD4 (top panel) and CD8 (bottom panel) T cell frequencies by combinational immunotherapeutics. Data shown are % of CD4 and CD8 T cells in the spleen on day 32 after the start of antibody treatment. **b** Representative FACS profiles depicting the increase of memory and effector CD4 (top panels) or CD8 (bottom panels) T cells in mice that received monotherapy and combination treatment of anti-PD-1 plus Ipilimumab during the perinatal period. **c**, **d** Summary data on the phenotype of CD4 **c** and CD8 **d** T cells in mice that received combination treatments with anti-PD-1 plus anti-CTLA-4 mAbs. Data shown are % of cells with phenotypes of naive, central memory and effector memory phenotypes. Data shown are summarized from four experiments involving 7–11 female mice and 2–6 male mice per group. Statistical tests used: **a**, one-way ANOVA with Bonferroni multiple comparison test; **c** and **d**, one-way ANOVA with Bonferroni multiple comparison test

To understand the mechanisms of severe AEs induced by Ipilimumab + anti-PD-1 combination therapy, we analyzed the frequency and functional subsets of T cells in three groups of mice that received control IgG, Ipilimumab + anti-PD-1 and L3D10 + anti-PD-1. The frequencies of CD4 and CD8 T cells in three groups were substantially the same (Fig. 4a). Using CD44 and CD62L markers, we observed a substantial expansion of effector memory T cells (CD44^hi^CD62L^lo^) in the Ipilimumab + anti-PD-1 group, although the frequency of central memory T cells (CD44^hi^CD62L^hi^) was unaffected (Fig. 4b, d). Correspondingly, the frequency of naive T cells was greatly reduced in the anti-PD-1 and Ipilimumab-treated groups (Fig. 4b, d). The abnormal T cell activation was not due to depletion of Treg cells, whose frequency was significantly elevated in the spleen (Supplementary information, Figure S6).

**Fig. 5:**
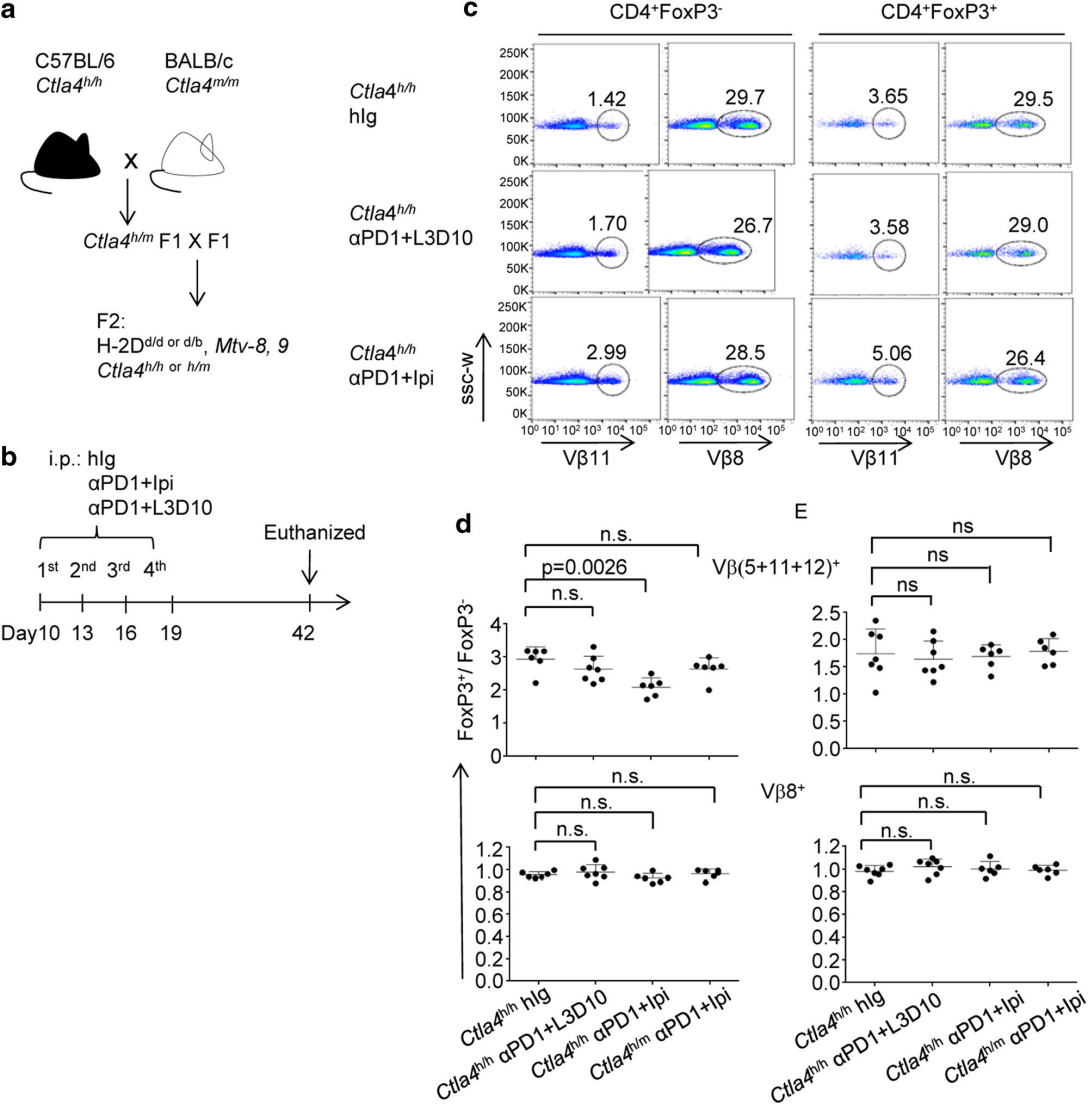
In combination with anti-PD-1, Ipilimumab preferentially expanded autoreactive Teff cells. **a** Diagram of the breeding scheme. **b** Diagram of the experimental timeline. The mice were produced in two steps. The first step was an outcross between two inbred strains as indicated. The second step was an intercross of F1s to obtain mice of designed genotypes (*H-2*^*d+*^*Ctla4*^*h/h or h/m*^*Mmtv*^*8+9+*^*)* for the studies. **c** Representative FACS profiles depicting the distribution of Vβ11, Vβ8 and Foxp3 markers among gated CD4 T cells from mice that received antibody treatments. **d** Composite ratios of Treg/Teff among VSAg-reactive (Vβ5^+^, 11^+^ or 12^+^, top panel) and non-reactive (Vβ8^+^) CD4 T cells. **e** Lack of impact on thymocytes. As in **d**, except the CD3^+^CD4^+^CD8^−^ thymocytes were analyzed. Data shown are means and S.D., *n* = 6–7 mice per group

In order to understand the pathogenesis of irAE, it is of critical importance to understand the impact of immunotherapy on autoreactive T cells. To address this issue, we took advantage of the fact that endogenous self-antigens are recognized by a few selective Vβs.^30^ Since C57BL/6 mice lack I–E to present endogenous superantigens, we generated F2 mice from (B6.*Ctla4*^*h/h*^ x BALB/c WT) F1×F1 cross and typed the offspring using mAbs that distinguish H-2^d^ (for the BALB/c background) and H-2^b^ (for the C57BL/6 background) haplotypes. We also used PCR of tail DNA to determine the status of mouse *Ctla4* vs human *CTLA4* alleles, as well as the endogenous VSAg8, 9 (Fig. 5a).

Using mice with a targeted mutation of *Ctla4*, Yamaguchi *et al*. showed that, CTLA-4 helps to convert Vβ5, 11 and 12-expressing T cells into Treg cells as targeted mutation of CTLA-4 increased the % of Teff.^31^ Therefore, we analyzed the impact of anti-PD-1 + Ipilimumab or anti-PD-1 + L3D10 on VSAg-reactive Teff and Treg cells in H-2^d+^ *CTLA4*^*h/h*^ mice (Fig. 5b). Representative data using Vβ11, which reacts with VSAg8, 9, are shown in Fig. 5c and summarized Treg/Teff ratios of VSAg-reactive T cells in Fig. 5d. The data for specific Vβ are provided in Supplementary information, Table S1. Ipilimumab + anti-PD-1 doubled the frequency of Foxp3^−^Vβ11^+^CD4 T cells but increased that of the Foxp3^+^Vβ11^+^CD4 T cells by merely 30% (Fig. 5c). Thus, Ipilimumab + anti-PD-1 not only increased the frequency of autoreactive T cells, but also reduced the frequency of Treg cells among the autoreactive T cells. The frequency of non-VSAg-reactive T cells (Vβ8^+^) was unaffected regardless of Foxp3 expression. In contrast, anti-PD-1 + L3D10 had no effect on the frequency of CD4 T cells. The selective expansion of VSAg-reactive Teff cells was also observed among Vβ5^+^ and Vβ12^+^ CD4 T cells (Supplementary information, Table S1). As a result, the Treg/Teff ratio among all studied VSAg-reactive CD4 T cells was significantly reduced in mice receiving anti-PD-1 + Ipilimumab (*P* = 0.0026). The reduction was selective for VSAg-reactive T cells as the Treg/Teff ratio among Vβ8+ cells was unaffected. These data demonstrate that antigen-specific suppression of autoreactive T cells is weakened by anti-PD-1 + Ipilimumab treatment. Furthermore, to address whether the treatment affected Treg/Teff during T cell development, we also analyzed the Treg/Teff ratio among VSAg-reactive thymocytes. As shown in Fig. 5e, anti-PD-1 + Ipilimumab had no impact on Treg/Teff ratio among thymocytes.

The anti-CTLA-4 mAbs used in this study react with human, but not mouse, CTLA-4 (Supplementary information, Figure S7) and thus cannot block the function of all CTLA-4 molecules in heterozygous mice carrying mouse *Ctla4* and human *CTLA4* alleles (*Ctla4*^*h/m*^). We tested if engaging a maximum of 50% of CTLA-4 is sufficient to cause reduced Treg/Teff among VSAg-reactive T cells. We found that in the *CTLA4*^*h/m*^ mice, there was no alteration in the ratio of conventional T cells to Treg cells regardless of antibody treatment.

### Humanized L3D10 clones exhibit potent CITE but minimal irAE

**Fig. 6:**
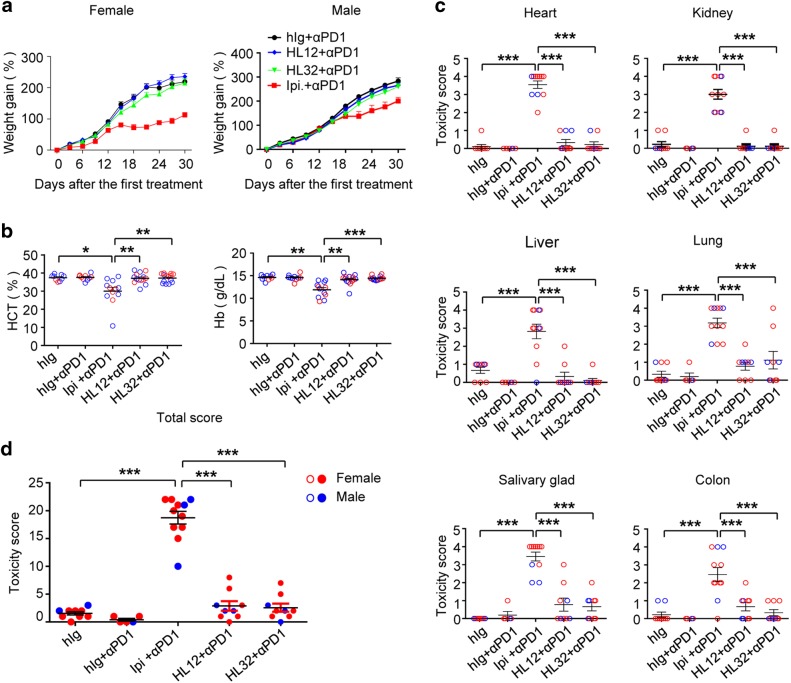
Humanized L3D10 clones maintained safety profiles when used in combination therapy with anti-PD-1 mAb. **a** Comparing humanized L3D10 clones HL12 and HL32 with Ipilimumab for their combination toxicity when used during perinatal period. Except changes in antibodies used, the experimental regimen was identical to that depicted in Fig. 1a. **b** Ipilimumab but not humanized L3D10 clones induced anemia when used in combination with anti-PD-1 antibody. **c** Pathology scores of internal organs and glands after the mice were treated with either control of given combination of immunotherapeutic drugs. **d** Composite pathology scores. Blue circles represent scores of male mice and the red scores represent female mice used. All scorings were performed double blind. Data are mean ± S.E.M., *n* = 5–12 mice per group. The samples were collected from five independent experiments and have been scored double blind. Statistical methods used were: **a** repeated measures two-way ANOVA with Bonferroni’s multiple comparison test; **b** non-parametric one-way ANOVA (Kruskal–Wallis test) with Dunn’s multiple comparisons test; **c** and **d** one-way ANOVA with Bonferroni’s multiple comparison test

As the first step to translate the L3D10 antibody into clinical testing, we humanized L3D10 and produced two clones with comparable binding to CTLA-4 that we compared to Ipilimumab for both irAE and CITE. In *Ctla4*^*h/h*^ mice, Ipilimumab but not HL12 and HL32 caused growth retardation when in combiation with anti-PD-1 (Fig. 6a). In contrast to Ipilimumab, neither HL12 nor HL32 induced anemia as measured by HCT and Hb (Fig. 6b). Histopathology analyses further confirmed that when combined with anti-PD-1, HL12 and HL32 induced no inflammation in heart, liver, colon or kidney, although moderate inflammation in lung and salivary glands was observed in a small proportion of mice (Fig. 6c). The composite pathology scores revealed that HL12 and HL32 induced even less inflammation than L3D10 in combination therapy (compare Figs. 6d and 3c). Furthermore, no systemic activation of T cells was induced by the humanized clones when used in combination with anti-PD-1 antibody (Supplementary information, Figure S8). Therefore, the safety profile of L3D10 was not compromised during humanization.

**Fig. 7:**
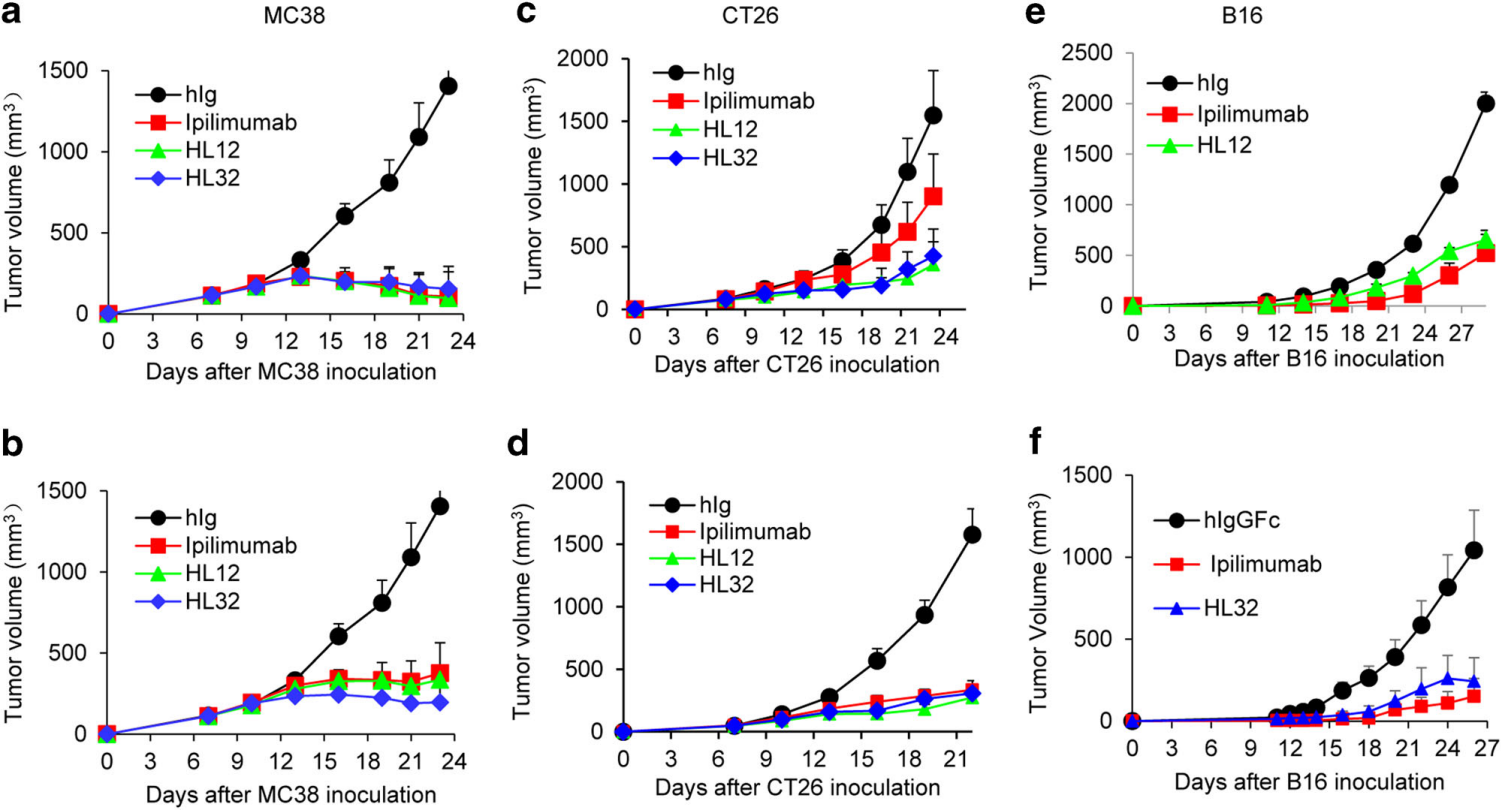
Comparison of the immunotherapeutic effect of HL12 and HL32 with Ipilimumab. **a**, **b** MC38-bearing-*Ctla4*^*h/m*^ mice (*n* = 5) were i.p. treated with 30 μg **a** or 10 μg **b** of either control hIg, Ipilimumab, HL12 or HL32 on day 7, 10, 13 and 16. **c**, **d** CT26 bearing-Ctla4^*h/m*^ mice (*n* = 6–10) were i.p. treated with 150 μg **c** or 100 μg **d** of either control Ig, Ipilimumab, HL12 or HL32 on day 7, 10, 13 and 16. **e**, **f** B16-bearing Ctla4^*h/h*^ mice (*n* = 5–6) were i.p. treated with 250 μg control Ig, Ipilimumab, HL12 **e** or HL32 **f** Data are mean ± S.E.M. and data were analyzed by repeated measures two-way ANOVA with Bonferroni’s multiple comparison test. In all settings, HL12 and HL32 induced statistically significant tumor rejection when compared with Control hIgG, HL12 (**a**, *P* = 0.0023; **b**, *P* = 0.0105; **c**, *P* *<* 0.0001; **d**, *P* = 0.0272; **e**, *P* *<* 0.0001); HL32 (**a**, *P* = 0.004; **b**, *P* = 0.0059; **c**, *P* *<* 0.0001; **d**, *P* = 0.0259; **f**, *P* = 0.1003). Tumor rejections induced by Ipilimumab were also significant in all but except one setting (**a**, *P* = 0.0026; **b**, *P* = 0.0231; **c**, *P* = 0.2; **d**, *P* = 0.0003, **e**, *P* = 0.0145; **f**, *P* = 0.0234). The differences between different therapeutic antibodies are not statistically significant

To determine whether better safety of HL12 and HL32 was achieved at the expense of therapeutic effect, we first compared Ipilimumab with HL12 and HL32 for their therapeutic effect. Previous studies by us and others have revealed that anti-murine CTLA-4 mAb monotherapy is capable of inducing rejection of MC38 colon cancer cells of C57BL/6 origin and CT26 cells of BALB/c origin. We therefore generated F1 mice (*Ctla4*^*h/m*^) by crossing BALB/c.*Ctla4*^*m/m*^ mice and C57BL/6.*Ctla4*^*h/h*^ mice. Whereas MC38 tumors grow unimpeded in the control Ig-treated mice, their growth was prevented by adding low doses of anti-human CTLA-4 mAbs (Fig. 7). When injected four times at 30 μg/injection, all anti-CTLA-4 mAbs were equally potent (Fig. 7a). When injected four times at a low dose of 10 μg/injection, HL32 appeared somewhat more potent than Ipilimumab and HL12, although the difference was not statistically significant (Fig. 7b). CT26 is somewhat more resistant than MC38 to anti-CTLA-4 immunotherapy, and thus requires higher doses. When high doses of antibodies were used, all three mAbs-induced statistically significant growth inhibition (Fig. 7c). At a lower dose, Ipilimumab did reduce tumor growth somewhat, although the reduction was not statistically significant (*P* = 0.29). On the other hand, both HL12 and HL32 induced clear growth inhibition (*P* *<* 0.001) (Fig. 7d). Significant inhibitions were achieved by both antibodies when B16-F10 melanoma tumor models were used (Fig. 7e, f). Taken together, the humanized L3D10 clones HL12 and HL32 are at least as potent as Ipilimumab in causing tumor rejection. Therefore, the humanized mouse model allowed us to identify dramatically safer but at least equally potent anti-CTLA-4 mAbs.

### In Ctla4^h/m^ mice, engagement of human CTLA-4 is sufficient for inducing tumor rejection but not for autoimmune disease

**Fig. 8:**
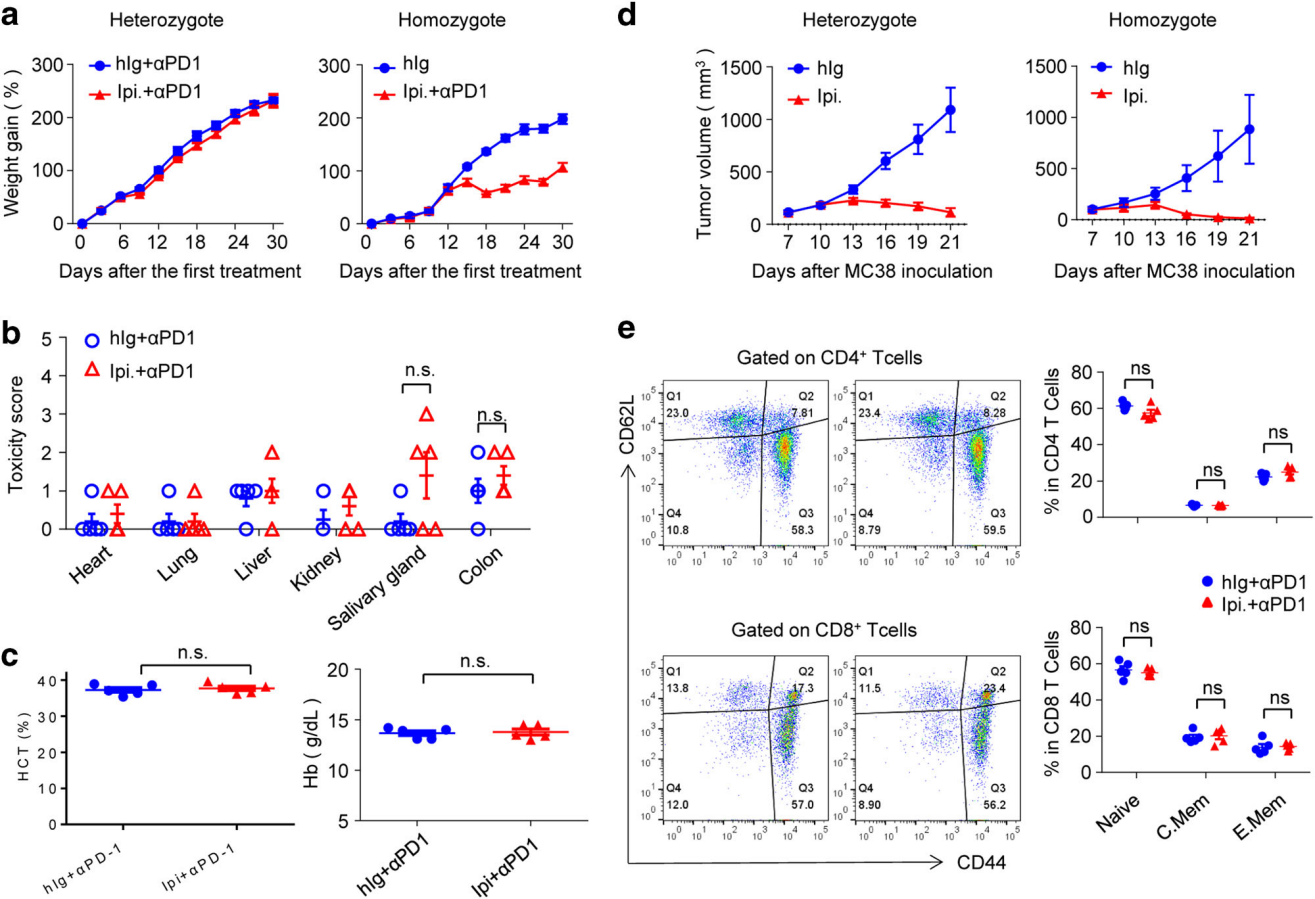
Distinct genetic requirement for irAE and CITE revealed in *C57BL/6.Ctla4*^*h/m*^ mice. **a**–**c** Evaluation of irAE. Female mice (*n* = 5) of given genotypes were treated with either control human IgG (hIg), or anti-PD-1 + Ipilimumab during the perinatal period and evaluated for body weight gain, inflammation and red blood cell anemia at 6 weeks of age. **a** Ipilimumab + anti-PD-1 combination induced growth retardation in *Ctla4*^*h/h*^ but not the *Ctla4*^*h/m*^ mice. **b** Except for a modest induction in some mice in the salivary gland, Ipilimumab + anti-PD-1 did not induce inflammation in internal organs in heterozygous mice. **c** Ipilimumab + anti-PD-1 did not induce red blood cell anemia in heterozygous mice. **d** Effective tumor rejection induced by Ipilimumab. Tumor-bearing *Ctla4*^*h/h*^ and *Ctla4*^*h/m*^ mice received treatment of either control hIg or Ipilimumab (100 μg/injection × 4) on days 7, 10, 13 and 16. The tumor growth was measured every 3 days. Data are mean ± S.E.M. and all Data shown have been reproduced two times. **e** Ipilimumab + anti-PD-1 did not cause systemic T cell activation in *Ctla4*^*h/m*^ mice. Representative FACS profiles depicting the distribution of CD44 and CD62L are shown on the left and summary data are shown on the right. Data in **a** and **d** were analyzed by repeated measures two-way ANOVA with Bonferroni’s multiple comparison test; whereas those in **b**, **c** and **e** were analyzed by unpaired two-tailed Student’s *t* test

The above data that Ipilimumab can induce tumor rejection in *CTLA4*^*h/m*^ mice raised an intriguing issue as to whether this mAb can induce irAE by engaging only part of the cell surface CTLA-4. Since anti-human CTLA-4 mAbs used in this study do not react with mouse CTLA-4 molecules (Supplementary information, Figure S7), we evaluated whether irAE and CITE require similar levels of receptor engagement by comparing irAE and CITE in *Ctla4*^*h/m*^ mice. Surprisingly, the same dose of Ipilimumab + anti-PD-1 that induced growth retardation in *Ctla4*^*h/h*^ mice (Fig. 1a) failed to do so in *Ctla4*^*h/m*^ mice (Fig. 8a), Consistent with the absence of irAE, histopathological analysis revealed that, with the exception of moderate inflammation in the salivary gland, anti-PD-1 + Ipilimumab did not cause inflammation in any other organs analyzed (Fig. 8b), even though the doses used caused severe inflammation in essentially all organs analyzed in homozygous mice (Figs. 2 and 3). Similarly, no anemia was observed in anti-PD-1 + Ipilimumab-treated *Ctla4*^*h/m*^ mice (Fig. 8c). Nevertheless, the heterozygous mice are nearly as responsive as the homozygous mice in respect to immunotherapy by Ipilimumab (Fig. 8d). Therefore irAE and cancer immunity can be uncoupled genetically: whereas the human *CTLA4* gene confers CITE responses to Ipilimumab in a dominant fashion, its role in conferring irAE is recessive. These data also suggest that distinct mechanisms are responsible for irAE vs CITE.

In contrast to what was observed in homozygous mice (Fig. 4), the combination of Ipilimumab + anti-PD-1 did not induce systemic activation of T cells in *Ctla4*^*h/m*^ mice (Fig. 8e). To understand the distinct autoimmune adverse effect, we analyzed the impact of anti-PD-1 + Ipilimumab on the Treg/Teff ratio in mice homozygous and heterozygous for human CTLA-4. The same treatment that reduced Treg/Teff ratio in such homozygous mice had no effect in heterozygous mice (Fig. 5c, d). This distinct genetic requirement further strengthens the notion that autoimmune adverse effect can be uncoupled from cancer immunity. The lack of systemic T cell activation and failure to selectively expand autoreactive Teff cells explains the lack of irAE in *Ctla4*^*h/m*^ mice.

### Observing irAE and CITE in the same setting

**Fig. 9:**
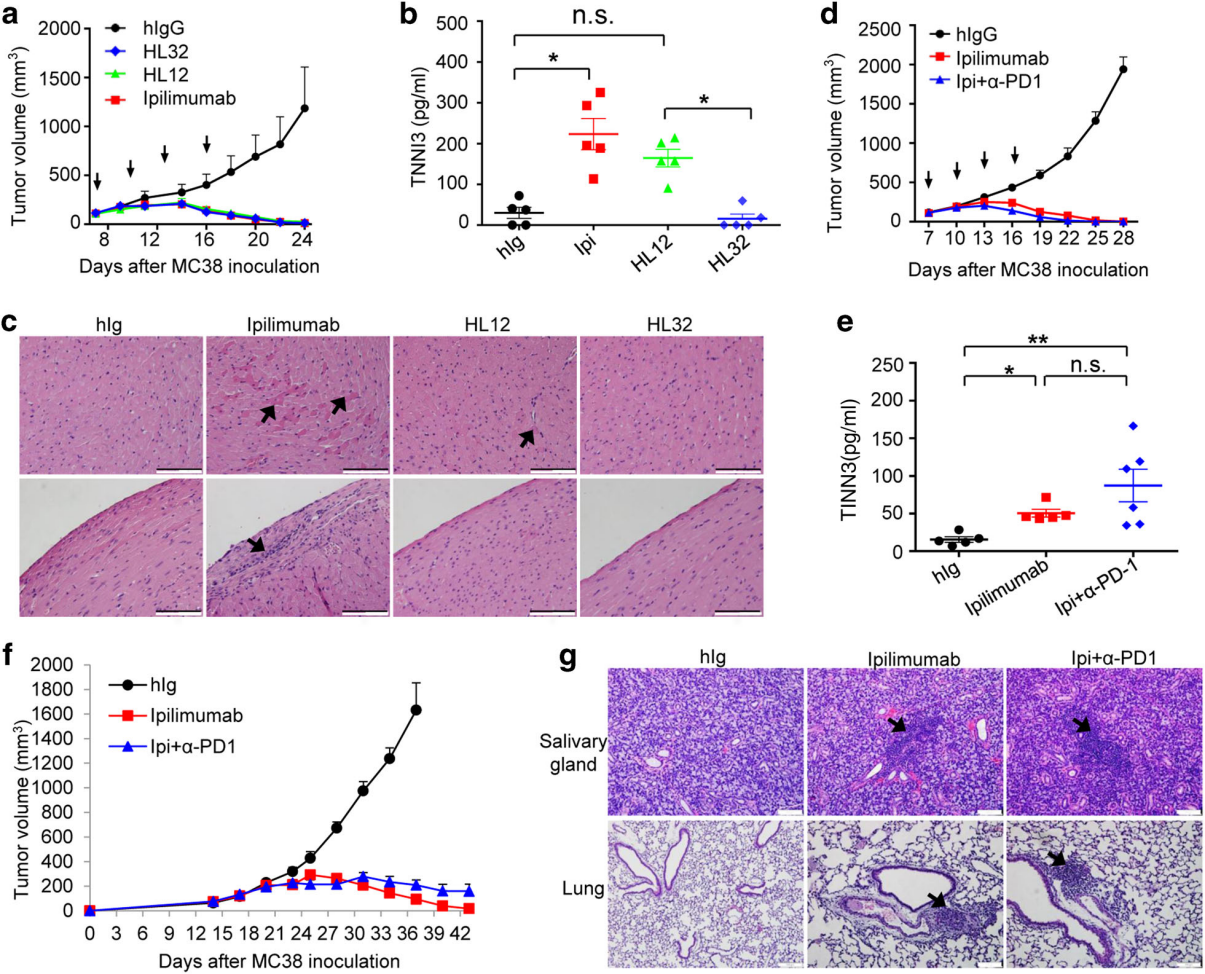
irAE and CITE in 6-7-week-old young adult and 10-day old tumor- bearing mice. **a**–**c** MC38-bearing young male mice (7-week-old) were inoculated with MC38 tumor cells and treated with either control hIgG, Ipilimumab, HL12 or HL32(100 μg/injection × 4) on days 7, 10, 13 and 16 after tumor cell challenges. **a** Tumor volumes over time. **b** Serum TNNI3 levels on day 25 after tumor challenge were determined by ELISA. **c** H&E staining show hyalinization and inflammation in myocardium. Scale bar 100 μm. **d**, **e** MC38-bearing young male mice (6-week-old) were inoculated with MC38 tumor cells and treated with either control hIgG, Ipilimumab or Ipilimumab + anti-PD-1(100 μg/injection × 4) on days 7, 10, 13 and 16 after tumor cell challenges. **d** Tumor volumes over time. **e** Serum TNNI3 levels on day 25 after tumor challenge were determined by ELISA. **f** 10-day-old mice were challenged with MC38 tumors, and immunotherapies were initiated on days 14, 17, 20 and 23 days of age and tumor sizes over time were presented. Data are mean ± S.E.M. and analyzed by repeated measures two-way ANOVA with Bonferroni’s multiple comparison test. hIg vs Ipilimumab or Ipi + anti-PD-1, *P* *<* 0.0001; Ipilimumab vs Ipi + α-PD-1, ns. **g** Combination therapy and monotherapy-induced multiple organ inflammations. Representative H&E sections from salivary gland and lung are presented. Scale bar, 100 μm. **b**, **e** Data are mean ± S.E.M. and statistical significance was analyzed by non-parametric one-way ANOVA

While we have so far used distinct, separate settings to allow more robust evaluation of irAE and CITE, it is of interest to show irAE and CITE can be observed in the same setting. We took two approaches to achieve this goal. First, we evaluated adverse events on the hearts of young adult mice receiving anti-CTLA-4 antibody treatment based on both levels of cardiac troponin I (TNNI3, a routine diagnostic marker for various heart disorders) as serum marker and histological analysis. As shown in Fig. 9a, anti-CTLA-4 mAbs-induced similarly robust tumor rejection in young (6–7-week-old) adult mice. Despite similar degrees of tumor rejection, the three mAbs-induced distinct adverse heart defects. Notably, Ipilimumab-induced high levels of TNNI3 (Fig. 9b) and histological analysis revealed extensive hyaline deposits within and outside myocytes (Fig. 9c, upper panel) with extensive pericardial inflammation (Fig. 9c, lower panel). Significant although lower levels of TNNI3 were observed in sera of HL12-treated mice, with correspondingly lower levels of hyalination and inflammation in the heart. In contrast, no elevation in serum TNNI3 and absence of either hyalination or inflammation were observed in HL32-treated mice. When combined with anti-PD-1, the therapeutic effect of Ipilimumab was comparable between monotherapy and combination therapy (Fig. 9d). Whereas toxicity to the heart was increased, there was no statistical significance between the group treated with Ipilimumab alone and the group treated with Ipilimumab plus anti-PD-1 due to high individual variations typical of toxicity studies (Fig. 9e).

Conversely, we tested CITE using 10-day-old mice as they were robust for evaluating irAE. MC38 tumors grew progressively after being transplanted into 10-day-old mice (Fig. 9f). Remarkably, the young mice were highly responsive to Ipilimumab both in tumor rejection and in induction of irAE, as demonstrated by rapid tumor regression (Fig. 9f) and pervasive servere organ inflammation (Fig. 9g).

### Systemic T cell activation strongly correlates with irAE

**Fig. 10:**
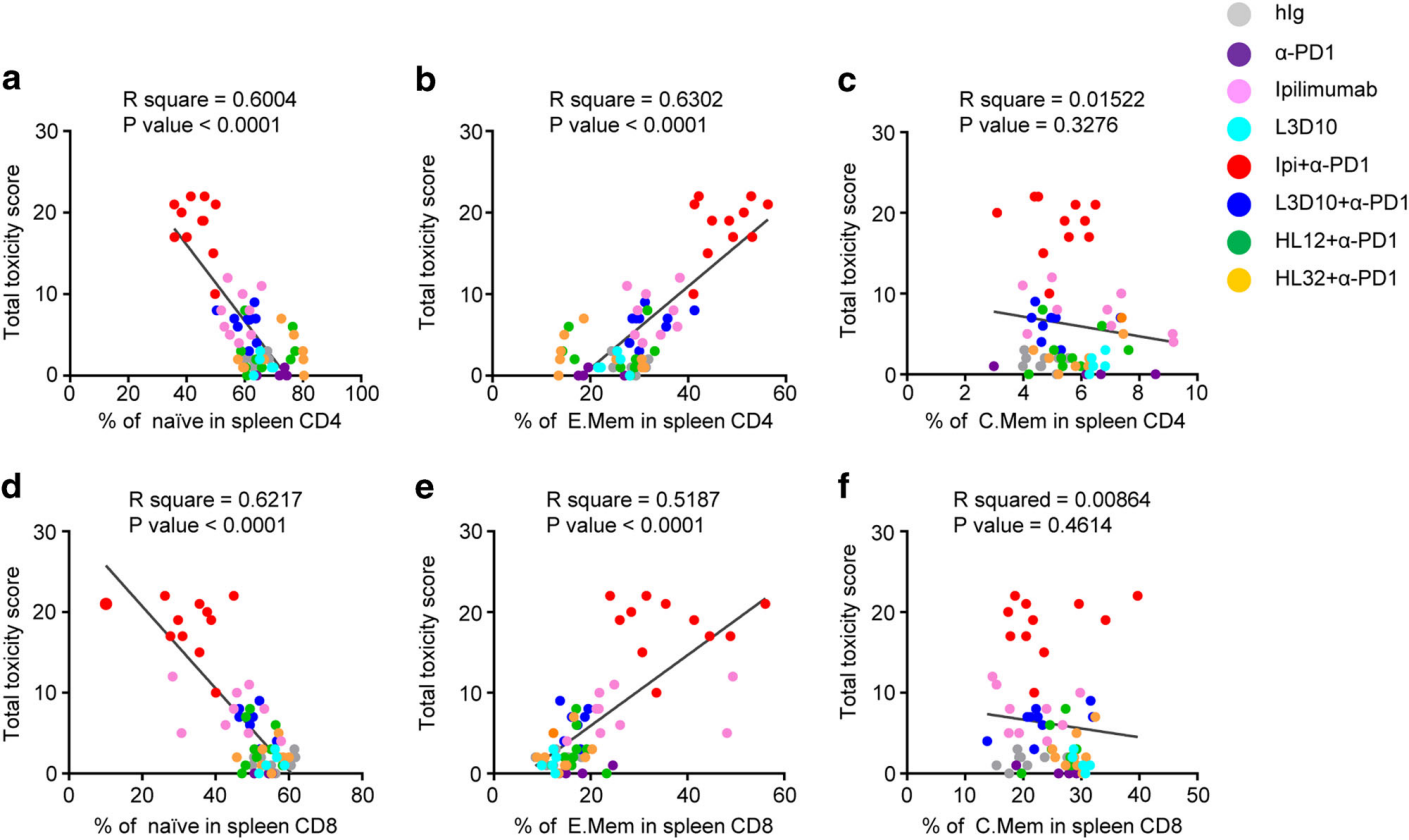
Loss of naive T cells and increase of effector memory T cells correlate with multiple organ inflammation. Data shown are re-analyses of data presented in Figs. 2, 3, 4, 6 and Supplementary information, Figure S8. Naive T cells: CD44^Lo^CD62L^Hi^ (**a** and **d**); effector memory T cells (E.Mem): CD44^Hi^CD62L^Lo^ (**b** and **e**); central memory T cells (C.Mem): CD44^Hi^CD62L^Hi^ (**c** and **f**). Correlation coefficient and *P*-value of linear regression were calculated by Pearson’s method

Since the various antibodies used in this study demonstrate distinctive profiles of irAE and peripheral T cell activation, it is of interest to determine whether peripheral T cell activation correlates with irAE. As shown in Fig. 10a, d, individual irAE scores of mice receiving either control or one of the five different anti-CTLA-4 mAbs administrations negatively correlate with the percentages of naive CD4 and CD8 T cells in the spleen. Percentages of central memory T cells do not show such correlation (Fig. 10c, f). In contrast, the percentage of effector memory T cells positively correlates with irAE (Fig. 10b, e). These strong correlations suggest that pervasive T cell activation in the periphery is potentially the underlying cause for irAE.

## Discussion

Since the description of irAE as a new clinical entity,^10^ there has been increasing interest in modeling the condition in mice in order to develop a means to overcome this major bottleneck for the advancement of cancer immunotherapy. Progress has been slow, however, perhaps because mouse tumor models differ from human cancer patients whose immune system has had chronic interactions with the cancer tissue. In addition, since irAE may well be drug-specific, it is difficult to model the irAE of a specific anti-human CTLA-4 mAb with an anti-mouse CTLA-4 mAb. Our study here used human *CTLA4* knock-in mice to evaluate irAE of clinically used anti-CTLA-4 mAb. We showed that this model successfully recapitulated most pathological observations associated with Ipilimumab, either alone or in combination with anti-PD-1 mAb, including severe inflammation to organs, such as heart, lung, liver, kidney and intestine. Rare diseases associated with Ipilimumab, such as pure red cell aplasia,^19,20^ were also observed in our model.

It should be noted that while our models can be used to mimic the combination of Ipilimumab and anti-PD-1, anti-PD-1 alone did not induce irAE in our model. Consistent with clinical observations, whereas Ipilimumab alone does induce significant adverse effects based on multiple organ inflammation, it is considerably less severe than combination therapy. Furthermore, in order to observe severe irAE, we had to use very young mice. However, while the adverse effect was less severe, we did observe heart disease (Fig. 9) and kidney destruction (Supplementary information, Figure S9). It is of interest to note that of the two humanized L3D10 clones, one appears safer than the other with respect to heart pathology. Overall, we observed improved safety after humanization without compromising efficacy (Supplementary information, Figure S10). Therefore, *Ctla4*^*h/h*^ mice can be used to discriminate highly similar antibodies and thus to select sub-clones for further clinical development. Our companion study demonstrates that humanization largely abrogated the blocking activity of L3D10 without compromising either therapeutic effect or safety, further suggesting that neither CITE nor irAE relates to the blockade of the CTLA-4-B7 interaction.

While it is better to use very young mice to evaluate irAE of anti-CTLA-4 mAbs, such mice also exhibit strong CITE after Ipilimumab treatment. Since many of the irAE, such as retarded growth and defective development of reproductive system, were observed in young mice, our model may be valuable in predicting potential irAE that are uniquely important for pediatric cancer patients.

It is established that due to lymphopenia, T cells undergo extensive homeostatic proliferation in young mice.^32,33^ Since cancer patients and young mice are often lymphopenic, and lymphopenia is associated with homeostatic proliferation and autoimmune diseases,^34,35^ it is of great interest to consider whether lymphopenia is a co-factor for the irAEs. If this is the case, one may use lymphopenia as a potential biomarker for irAE. Furthermore, our data demonstrated that tumor-bearing mice resemble young mice in expressing higher levels of *Ctla4*. Therefore, data from young mice may shed light on tumor-bearing hosts. The spectrum of organ inflammation, including cardiomyoditis, aplastic anemia and endocrinopathy in the young mice recapitulates clinical findings and lends strong support for this thesis.

Liu *et al*. have recently used partial Treg depletion to sensitize mice for irAE.^36^ While this model recapitulated some pathological features of irAE, it is noted that Ipilimumab systematically expands rather than depletes Treg cells in human cancer patients,^37^ a feature we observed when we used Ipilimumab in our human *CTLA4* knock-in mice (data not shown). For this reason, it is unlikely that a Treg depletion-based model reflects the cause of irAE in cancer patients. Nevertheless, since we found that combination therapy reduced the Treg/Teff ratios, a general defect in Treg may recapitulate some pathological features of irAE.

Using mice that are either homozygous or heterozygous for human *CTLA4* alleles, we were able to genetically uncouple irAE and CITE. Thus, while irAE is observed only in homozygous mice, CITE is observed in both heterozygous and homozygous mice. The marked difference in genetic requirement suggests distinct mechanisms for irAE and CITE: whereas irAE represents loss of CTLA-4 function imposed by Ipilimumab, CITE represents a gain of function of human *CTLA-4* gene.

**Fig. 11:**
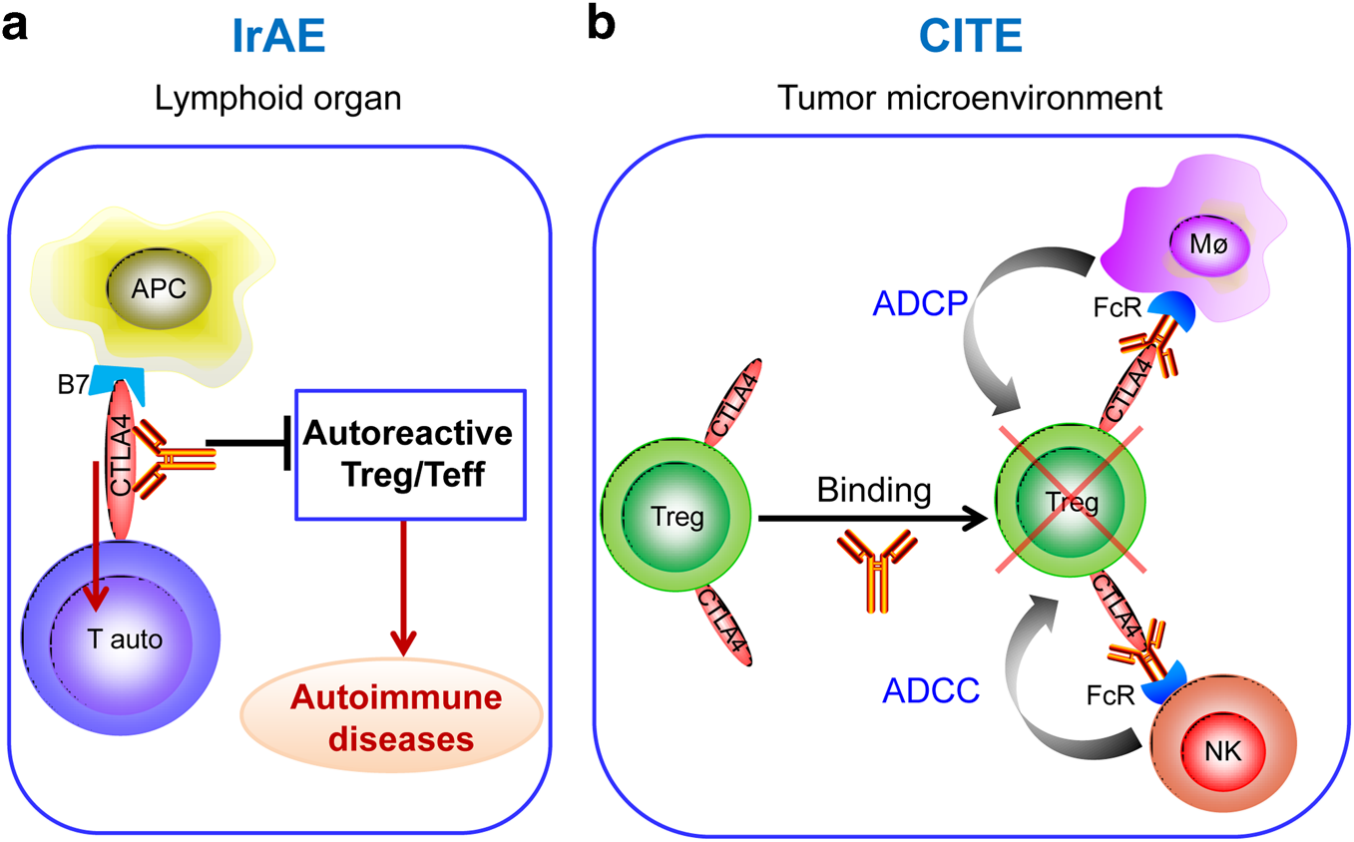
Distinct mechanisms responsible for irAE and CITE. **a** irAE is caused by inhibiting the conversion of autoreactive T cells into autoreactive Treg cells, which leads to a polyclonal expansion of autoreactive T cells in the peripheral lymphoid organs. **b** Tumor rejection is achieved by FcR-mediated depletion of Treg cells in the tumor microenvironment and is independent of naive T cell activation in the peripheral lymphoid organs. Neither irAE nor CITE depends on blockade of B7-CTLA-4 interaction

Immunologically, the distinct genetic requirement is reflected by general T cell activation because Ipilimumab + anti-PD-1-induced extensive T cell activation in homozygous mice but not heterozygous mice. Using endogenous superantigen-reactivity as the marker for autoreactivity, we found that Ipilimumab + anti-PD-1 prevented conversion of autoreactive T cells into Treg cells resulting in an increased ratio of autoreactive effector cells over autoreactive Treg cells. Our previous studies demonstrated that Treg cells are the most effective in suppressing T cell activation in vivo if they shared the antigen-specificity with the effector T cells.^38^ Therefore, the increased ratio of autoreactive effector to autoreactive Treg cells appears to allow activation of autoreactive T cells, leading to autoimmune diseases, as proposed in Fig. 11a.

It has been demonstrated that bi-allelic deletion of the *CTLA4* gene reduced conversion of autoreactive T cells into Treg cells.^31^ The requirement for bi-allelic engagement by anti-CTLA4 mAbs for irAE is at least partially explained by the requirement for bi-allelic engagement of CTLA-4 in the conversion, as an increased ratio of autoreactive effector/regulatory T cells could lead to autoimmune diseases. The convergence between genetic inactivation of the *Ctla4* locus and bi-allelic antibody engagement raised the intriguing possibility that Ipilimumab somehow inactivated the CTLA4 molecules. Since our companion paper demonstrated that Ipilimumab does not block the B7-CTLA-4 interaction under physiological conditions, the mechanism by which Ipilimumab inactivates CTLA-4 molecules remains to be determined.

Consistent with a dominant function of human CTLA-4 in CITE, several recent studies including those of our own have demonstrated a critical role for local depletion of Treg cells in the tumor microenvironment. Thus, using anti-mouse CTLA-4 mAbs with identical Fv but distinct isotypes of Fc, Selby *et al*. demonstrated that the ability of anti-mouse CTLA-4 mAbs to induce tumor rejection is determined by the Fc portion.^16^ Specifically, those with stronger affinity for activating FcgRs, including IgG2a and IgG2b can effectively induce tumor rejection and Treg depletion in the tumor microenvironment. In contrast, those with weaker affinity failed to do so. Consistent with this notion, Bulliard *et al*.^18^ showed that the Fcer1 gene, which encodes the activating signaling receptor subunit, is essential for anti-CTLA-4 mAb-induced tumor rejection. Furthermore, among the activating FcγRs that incorporate the Fcer1-encoded subunits, Simpson *et al* showed that tumor rejection and Treg depletion requires engagement of activating FcγRIV,^17^ suggesting an obligatory interaction between the Fc portion of anti-CTLA-4 mAb and FcR on either neutrophils or macrophages. Our data presented in the companion paper further demonstrate that anti-CTLA-4-induced tumor rejection requires Treg depletion but not blockade of the B7-CTLA-4 interaction (Fig. 11b). Since the CTLA-4 mAbs were comparable in tumor rejection, but yet vary greatly in inducing peripheral T cell activation, our data are inconsistent with the notion that anti-CTLA-4 antibodies promote tumor rejection by stimulating naive T cell activation in the periphery.^1^ The distinct mechanism and locality associated with irAE and CITE provide new insights on ways to produce more effective and safer CTLA-4-targeting reagents that favor Treg depletion within the tumor microenvironment while avoiding general T cell activation in the peripheral lymphoid organs.

The classical checkpoint blockade hypothesis has suggested that anti-CTLA-4 mAb induces tumor rejection by inducing activation of naive T cells in the lymphoid organs. In contrast, our data showed that it is actually the ability of mAbs to cause general activation of T cells in the lymphoid organs that correlates with irAE rather than CITE. This is highlighted by the striking correlations between the irAE score and systemic T cell activation triggered by combination therapy. In contrast, our companion paper demonstrated that Ipilimumab can induce tumor rejection without the de novo priming of antigen-specific T cells. This is because at the time of Ipilimumab treatment, priming of T cells has already been achieved. At this point, release of local suppression by Treg, rather than T cell priming in the lymphoid organs becomes the key to unleash cancer immunity.

Taken together, our work addresses the fundamental issue of whether irAE and CITE can be uncoupled to allow development of safer and more effective immunotherapeutic antibodies. We have described a new model that faithfully recapitulates irAEs and, using this model, we demonstrate that irAE and CITE are not inherently linked. This concept provides a foundation to identify therapeutic anti-CTLA-4 mAbs that are at least as effective as, but significantly less toxic than Ipilimumab. Our data demonstrate that humanized L3D10 clones are potential candidates for therapeutic development for human cancer therapy. The notion that T cell activation in the tumor microenvironment entails cancer immunity, while general T cell activation in the peripheral lymphoid organs risks autoimmunity (Fig. 11) is likely to have broad implications for the selection of targets as well as targeting therapeutic candidates.

## Materials and Methods

### Animals

*CTLA4* humanized mice that express the CTLA-4 protein with 100% identity to human CTLA-4 protein under the control of the endogenous mouse *Ctla4* locus have been described.^24^ The homozygous knock-in mice (*Clta4*^*h/h*^) were backcrossed to the C57BL/6 background for at least 10 generations. Heterozygous mice (*Ctla4*^*h/m*^) were produced by crossing the *CTLA4*^*h/h*^ mice with either wild type (WT) BALB/c mice (for tumor growth studies) or WT C57BL/6 mice (for irAE studies). WT BALB/c and C57BL/6 mice were purchased from Charles River Laboratories through an NCI contract. All mice were maintained at the Research Animal Facility of the Children’s Research Institute at the Children’s National Medical Center. All studies involving mice have been approved by the Institutional Animal Care and Use Committee.

### Cell culture

The murine colon tumor cell line, MC38, was described previously,^2^ and CT26 and B16-F10 cell lines were purchased from the ATCC (Manassas, VA, USA). After receipt from the vendors, cell passages were kept minimal before in vivo testing. Cell lines were neither authenticated nor regularly tested for mycoplasma contamination. MC38, CT26 and B16-F10 cell lines were incubated at 37 °C with 5% CO_2_. MC38 and B16 cells were grown in DMEM (Dulbecco’s Modified Eagle Medium, Gibco) supplemented with 10% FBS (Hyclone), 100 units/ml of penicillin and 100 μg/ml of streptomycin (Gibco). CT26 cells were cultured in complete RPMI 1640 Medium (Gibco).

### Antibodies

Mouse anti-human CTLA-4 mAb L3D10 has been described.^28^ Anti-CTLA-4 mAb L3D10 used in the study was a chimeric antibody consisting of human IgG1 Fc and the variable regions of L3D10. Recombinant antibody was produced by Lakepharma, Inc (Belmont, CA, USA) through a service contract. Recombinant Ipilimumab with the amino acid sequence disclosed in WC500109302 and http://www.drugbank.ca/drugs/DB06186 was provided by Alphamab Inc. (Suzhou, Jiangsu, China), and Lakepharma Inc. (San Francisco, CA, USA). Clinically used drug was also used to validate the key results. Human IgG-Fc (no azide) was bulk ordered from Athens Research and Technology (Athens, GA, USA). Anti-mouse PD-1 mAb RMP1-14 was purchased from Bio-X Cell, Inc. (West Lebanon, NH, USA). Endotoxin levels of all mAbs were determined by LAL assay (Sigma) and were lower than 0.02 EU/μg.

### Tumor growth and regression assay

Mice with either heterozygous or homozygous knock-in of human *CTLA4* gene were challenged with given numbers of either the MC38 or CT26 colorectal cancer cell lines or the melanoma cell line, B16-F10. Immunotherapies were initiated at 2, 7 or 11 days after injection of tumor cells with indicated doses. The tumor growth and regression were determined using volume as the readout. The volumes (*V*) were calculated using the following formula.

*V* = *ab*^2^/2, where *a* is the long diameter, while *b* is the short diameter.

### Humanization of L3D10

The L3D10 antibody was humanized by Lakepharma, Inc. through a service contract. The first humanized chain for each utilizes a first framework and contains the most human sequence with minimal parental antibody framework sequence (Humanized HC 1 and LC 1). The second humanized chain for each uses the same framework as HC 1 and LC 1 but contains additional parental L3D10 antibody sequences (Humanized HC 2 and LC 2). The third humanized chain for each utilizes a second framework and, similar to HC 2/LC 2, also contains additional parental sequences fused with the human framework (Humanized HC 3 and LC 3). The three light and three heavy humanized chains were then combined in all possible combinations to create nine variant humanized antibodies that were tested for their expression level and antigen binding affinity to identify antibodies that perform similar to the parental L3D10 antibody.

### Complete blood counts

Blood samples (50 μl) were collected at 41 days of age using tubes with K_2_EDTA (BD) and analyzed by HEMAVET HV950 (Drew Scientific Group, Miami Lakes, FL, USA) following the manufacture’s manual.

### Histopathology analysis of internal organs

H&E sections were prepared from formalin-fixed organs harvested from mice that received therapeutic or control antibodies and were scored double blind. Score criteria: heart, infiltration in pericardium, right or left atrium, base of aorta, and left or right ventricle each count as 1 point; lung scoring is based on lymphocyte aggregates surrounding bronchiole, 1 stands for 1–3 small foci of lymphocyte aggregates per section, 2 stands for 4–10 small foci or 1–3 intermediate foci, 3 stands for more than 4 intermediate or presence of large foci, 4 stands for marked interstitial fibrosis in parenchyma and large foci of lymphocyte aggregates; liver scoring is based on lymphocyte infiltrate aggregates surrounding portal triad, 1 stands for 1–3 small foci of lymphocyte aggregates per section, 2 stands for 4–10 small foci or 1–3 intermediate foci, 3 stands for 4 or more intermediate or the presence of large foci, 4 stands for marked interstitial fibrosis in parenchyma and large foci of lymphocyte aggregates; kidney scoring: (1) mild increase of glomerular cellularity; (2) increase of glomerular cellularity and lymphocyte infiltration in distal or proximal tubes; (3) large lymphocyte aggregates in collecting ducts; (4) marked lymphocyte aggregates within cortex and medulla of kidney. Salivary gland scoring is based on lymphocyte infiltration in submandibular gland: 1 stands for 1–3 small foci of lymphocyte aggregates per section, 2 stands for 4–10 small foci or 1–3 intermediate foci, 3 stands for 4 or more intermediate or presence of large foci, 4 stands for marked interstitial fibrosis and tissue destruction in parenchyma and large foci of lymphocyte aggregates. Data shown are combined scores of all organs examined.

### Analysis of autoreactive T cells through F1 intercross

As diagrammed in Fig. 5a, we outcrossed the C57BL/6.*Ctla4*^*h/h*^ mice to WT BALB/c mice. The F1 mice were intercrossed to generate the F2 in which both the *Ctla4*^*h*^ and H-2 alleles randomly segregated. The *Ctla4* alleles and endogenous VSAg *Mmtv8, 9* were genotyped using tail DNA according to published reports,^24,30^ while the existence of H-2d haplotypes was determined by flow cytometry using peripheral blood leukocytes.

### Clinical chemistry for drug toxicity

The kit for measuring serum troponin i type 3, Cardiac (TNNI3) was purchased from Cloud-Clone Corp. (Cat. No. SEA478Mu), and TNNI3 levels were measured using ELISA following the manufacturer’s protocol. Creatinine levels were measured using a Creatinine (serum) Colorimetric Assay Kit (Cayman Chemical) or a Creatinine (CREA) Kit (RANDOX, Cat No, CR2336). Serum BUN levels were measured using a UREA NITROGEN DIRECT kit (Stanbio laboratory) according to the manufacture’s manual.

### Biostatistics

The specific tests used to analyze each set of experiments are indicated in the figure legends. For each statistical analysis, appropriate tests were selected on the basis of whether the data with outlier deletion was normally distributed by using the D’Agostino & Pearson normality test. Data were analyzed using an unpaired two-tailed Student’s *t* test or a Mann–Whitney test to compare between two groups, one-way analysis of variance (ANOVA) or Kruskal–Wallis test for multiple comparisons, and two-way repeated measures ANOVA for behavioral tests. The correlation coefficient and *P*-value of linear regression were calculated by Pearson’s method. Sample sizes were chosen with adequate statistical power on the basis of the literature and past experience. No samples were excluded from the analysis, and experiments were not randomized except where specified. Blinding was not done during animal group allocation but was done for some measurements made in the study (i.e., tumor size measuring, scoring of histology). In the graphs, *y*-axis error bars represent S.E.M. or S.D. as indicated. Statistical calculations were performed using Excel (Microsoft), GraphPad Prism software (GraphPad Software, San Diego, California) or R Software (https://www.r-project.org/). *P < 0.05, ** *P* *<* 0.01, *** *P* *<* 0.001.

### Data availability

All data generated or analyzed during this study are included in this article and its Supplementary information files. No other datasets were generated or analyzed during the current study.

## Electronic supplementary material

Supplementary information Table S1

Supplementary information Figure S1

Supplementary information Figure S2

Supplementary information Figure S3

Supplementary information Figure S4

Supplementary information Figure S5

Supplementary information Figure S6

Supplementary information Figure S7

Supplementary information Figure S8

Supplementary information Figure S9

Supplementary information Figure S10

## References

[1] Leach DR, Krummel MF, Allison JP (1996). Enhancement of antitumor immunity by CTLA-4 blockade. Science.

[2] Kocak E, Lute K, Chang X (2006). Combination therapy with anti-CTL antigen-4 and anti-4-1BB antibodies enhances cancer immunity and reduces autoimmunity. Cancer Res..

[3] Mokyr MB, Kalinichenko T, Gorelik L, Bluestone JA (1998). Realization of the therapeutic potential of CTLA-4 blockade in low-dose chemotherapy-treated tumor-bearing mice. Cancer Res..

[4] Hodi FS, O’Day SJ, McDermott DF (2010). Improved survival with ipilimumab in patients with metastatic melanoma. N. Engl. J. Med..

[5] Phan GQ, Yang JC, Sherry R (2003). Cancer regression and autoimmunity induced by cytotoxic T lymphocyte-associated antigen-4 blockade in patients with metastatic melanoma. Proc. Natl Acad. Sci. USA.

[6] Wolchok JD, Kluger H, Callahan MK (2013). Nivolumab plus Ipilimumab in advanced melanoma. N. Engl. J. Med..

[7] Larkin J, Chiarion-Sileni V, Gonzalez R (2015). Combined Nivolumab and Ipilimumab or monotherapy in untreated melanoma. N. Engl. J. Med..

[8] Hellmann MD, Rizvi NA, Goldman JW (2017). Nivolumab plus Ipilimumab as first-line treatment for advanced non-small-cell lung cancer (CheckMate 012): results of an open-label, phase 1, multicohort study. Lancet Oncol..

[9] Antonia S, Goldberg SB, Balmanoukian A (2016). Safety and antitumour activity of durvalumab plus tremelimumab in non-small cell lung cancer: a multicentre, phase 1b study. Lancet Oncol..

[10] Fecher LA, Agarwala SS, Hodi FS, Weber JS (2013). Ipilimumab and its toxicities: a multidisciplinary approach. Oncologist.

[11] Ribas A, Kefford R, Marshall MA (2013). Phase III randomized clinical trial comparing tremelimumab with standard-of-care chemotherapy in patients with advanced melanoma. J. Clin. Oncol..

[12] Beer TM, Kwon ED, Drake CG (2017). Randomized, double-blind, phase III trial of ipilimumab versus placebo in asymptomatic or minimally symptomatic patients with metastatic chemotherapy-naive castration-resistant prostate cancer. J. Clin. Oncol..

[13] Weber, J., et al. Adjuvant Nivolumab versus Ipilimumab in resected stage III or IV melanoma. *N. Engl. J. Med*. **377**, 1824–1835(2017). 10.1056/NEJMoa1709030 28891423

[14] Schadendorf D, Hodi FS, Robert C (2015). Pooled analysis of long-term survival data from phase II and phase III trials of ipilimumab in unresectable or metastatic melanoma. J. Clin. Oncol..

[15] Korman AJ, Peggs KS, Allison JP (2006). Checkpoint blockade in cancer immunotherapy. Adv. Immunol..

[16] Selby MJ, Engelhardt JJ, Quigley M (2013). Anti-CTLA-4 antibodies of IgG2a isotype enhance antitumor activity through reduction of intratumoral regulatory T cells. Cancer Immunol. Res..

[17] Simpson TR, Li F, Montalvo-Ortiz W (2013). Fc-dependent depletion of tumor-infiltrating regulatory T cells co-defines the efficacy of anti-CTLA-4 therapy against melanoma. J. Exp. Med..

[18] Bulliard Y, Jolicoeur R, Windman M (2013). Activating Fc gamma receptors contribute to the antitumor activities of immunoregulatory receptor-targeting antibodies. J. Exp. Med..

[19] Nair R, Gheith S, Nair SG (2016). Immunotherapy-associated hemolytic anemia with pure red-cell aplasia. New Engl. J. Med.

[20] Gordon IO, Wade T, Chin K, Dickstein J, Gajewski TF (2009). Immune-mediated red cell aplasia after anti-CTLA-4 immunotherapy for metastatic melanoma. Cancer Immunol. Immunother..

[21] Friedman CF, Proverbs-Singh TA, Postow MA (2016). Treatment of the immune-related adverse effects of immune checkpoint inhibitors: a review. JAMA Oncol..

[22] Bertrand A, Kostine M, Barnetche T, Truchetet ME, Schaeverbeke T (2015). Immune related adverse events associated with anti-CTLA-4 antibodies: systematic review and meta-analysis. BMC Med..

[23] Chen TW, Razak AR, Bedard PL, Siu LL, Hansen AR (2015). A systematic review of immune-related adverse event reporting in clinical trials of immune checkpoint inhibitors. Ann. Oncol..

[24] Lute K. D., et al. Human CTLA-4-knock-in mice unravel the quantitative link between tumor immunity and autoimmunity induced by anti-CTLA-4 antibodies. *Blood* **106**, 3127–3133 (2005). 10.1182/blood-2005-06-2298 PMC1895337 16037385

[25] Tivol EA (1995). Loss of CTLA-4 leads to massive lymphoproliferation and fatal multiorgan tissue destruction, revealing a critical negative regulatory role of CTLA-4. Immunity.

[26] Waterhouse P, Penninger JM, Timms E (1995). Lymphoproliferative disorders with early lethality in mice deficient in Ctla-4. Science.

[27] Klocke K, Sakaguchi S, Holmdahl R, Wing K (2016). Induction of autoimmune disease by deletion of CTLA-4 in mice in adulthood. Proc. Natl Acad. Sci. USA.

[28] May KF, Roychowdhury S, Bhatt D (2005). Anti-human CTLA-4 monoclonal antibody promotes T cell expansion and immunity in a hu-PBL-SCID model: a new method for preclinical screening of costimulatory monoclonal antibodies. Blood.

[29] Nishimura H, Okazaki T, Tanaka Y (2001). Autoimmune dilated cardiomyopathy in PD-1 receptor-deficient mice. Science.

[30] Abe R, Foo-Phillips M, Hodes RJ (1991). Genetic analysis of the Mls system. Formal Mls typing of the commonly used inbred strains. Immunogenetics.

[31] Yamaguchi T, Kishi A, Osaki M (2013). Construction of self-recognizing regulatory T cells from conventional T cells by controlling CTLA-4 and IL-2 expression. Proc. Natl Acad. Sci. USA.

[32] min B, Foucras G, Meier-Schellersheim M, Paul WE (2004). Spontaneous proliferation, a response of naive CD4 T cells determined by the diversity of the memory cell repertoire. Proc. Natl Acad. Sci. USA.

[33] min B (2003). Neonates support lymphopenia-induced proliferation. Immunity.

[34] King C, Ilic A, Koelsch K, Sarvetnick N (2004). Homeostatic expansion of T cells during immune insufficiency generates autoimmunity. Cell.

[35] Liu Y, Zheng P (2007). CD24: a genetic checkpoint in T cell homeostasis and autoimmune diseases. Trends Immunol..

[36] Liu J, Blake SJ, Harjunpaa H (2016). Assessing immune-related adverse events of efficacious combination immunotherapies in preclinical models of cancer. Cancer Res..

[37] Maker AV, Attia P, Rosenberg SA (2005). Analysis of the cellular mechanism of antitumor responses and autoimmunity in patients treated with CTLA-4 blockade. J. Immunol..

[38] Chang X, Zheng P, Liu Y (2009). Selective elimination of autoreactive T cells in vivo by the regulatory T cells. Clin. Immunol..

