# Supplementary material for: Uncoupling therapeutic from immunotherapy-related adverse effects for safer and effective anti-CTLA-4 antibodies in CTLA4 humanized mice
Source: Cell Res. 2018 Feb 20;28(4):433–47. doi: 10.1038/s41422-018-0012-z (PMC5939041; doi:10.1038/s41422-018-0012-z)
Supplement: Supplementary file 1 — Supplementary information Table S1 [file 41422_2018_12_MOESM1_ESM.pdf]

Supplemental information

Table S1. Ipilimumab induced preferential expansion of Foxp3$^-$ compartment among VSAg-reactive CD4 T cells

Group	Mice ID	Percentage of Vβs$^+$ in CD4$^+$FoxP3$^+$ or CD4$^+$FoxP3$^-$ (%)							
		CD4$^+$FoxP3$^-$				CD4$^+$FoxP3$^+$			
		Vβ11$^+$	Vβ12$^+$	Vβ5$^+$	Vβ8$^+$	Vβ11$^+$	Vβ12$^+$	Vβ5$^+$	Vβ8$^+$
Ctla4$^{h/h}$ hIg	6	1.64	0.14	0.04	36.00	3.79	1.26	0.74	33.20
	24	1.84	0.27	0.13	28.80	3.53	1.52	1.41	27.60
	29	1.42	0.23	0.16	29.70	3.65	1.40	0.68	29.50
	32	1.50	0.18	0.06	29.50	3.41	1.31	0.82	28.70
	35	1.77	0.21	0.12	27.10	3.69	1.07	1.50	25.30
	42	2.05	0.14	0.31	35.60	3.45	1.27	0.82	33.20
	MEAN±SD	1.70±0.23	0.20±0.05	0.14±0.10	31.12±3.74	3.59±0.15	1.31±0.15	1.00±0.36	29.58±3.14
Ctla4$^{h/h}$ α-PD1+L3D10	41	1.44	0.23	0.10	29.80	2.69	1.39	0.66	28.60
	44	1.25	0.12	0.04	29.90	3.15	1.11	0.40	30.30
	45	1.93	0.22	0.08	34.20	3.82	0.95	0.41	34.50
	53	1.70	0.18	0.16	26.70	3.58	0.96	0.80	29.00
	65	1.59	0.28	0.07	32.10	3.81	0.97	0.97	28.20
	71	2.24	0.28	0.14	29.40	3.68	1.42	0.72	27.80
	79	1.73	0.19	0.08	29.30	3.15	0.96	0.58	27.40
	MEAN±SD	1.70±0.32	0.21±0.06	0.09±0.04	30.20±2.37	3.41±0.42	1.11±0.21	0.65±0.21	29.40±2.44
Ctla4$^{h/h}$ α-PD1+Ipi	64	1.61	0.22	0.10	30.20	3.13	1.05	0.65	29.90
	73	2.99	0.47	0.15	28.50	5.06	2.11	0.81	26.40
	75	2.51	0.22	0.21	29.20	3.70	1.87	0.74	27.40
	81	2.82	0.30	0.23	28.00	3.85	0.88	1.41	24.90
	100	2.12	0.42	0.20	28.50	3.61	1.08	1.14	26.30
	101	3.10	0.52	0.44	35.00	3.89	1.48	1.60	30.60
	MEAN±SD	2.52±0.57**	0.36±0.13**	0.22±0.12	29.20±2.61	3.87±0.64	1.41±0.50	1.06±0.39	27.58±2.22
Ctla4$^{h/m}$ α-PD1+Ipi	8	1.58	0.22	0.09	27.10	2.90	1.05	1.12	25.50
	17	1.11	0.16	0.09	26.80	2.44	0.91	0.74	23.70
	33	1.80	0.22	0.11	29.10	3.46	1.35	0.93	28.90
	34	1.53	0.19	0.08	28.10	2.89	1.18	0.82	27.30
	61	1.32	0.13	0.05	29.10	2.92	0.78	0.41	28.70
	63	2.45	0.17	0.11	33.00	3.77	1.14	0.55	32.70
	MEAN±SD	1.63±0.46	0.18±0.04	0.09±0.02	28.87±2.24	3.06±0.47	1.07±0.20	0.76±0.26	27.80±3.11
