# Supplementary material for: Uncoupling therapeutic from immunotherapy-related adverse effects for safer and effective anti-CTLA-4 antibodies in CTLA4 humanized mice
Source: Cell Res. 2018 Feb 20;28(4):433–47. doi: 10.1038/s41422-018-0012-z (PMC5939041; doi:10.1038/s41422-018-0012-z)
Supplement: Supplementary file 2 — Supplementary information Figure S1 [file 41422_2018_12_MOESM2_ESM.pdf]

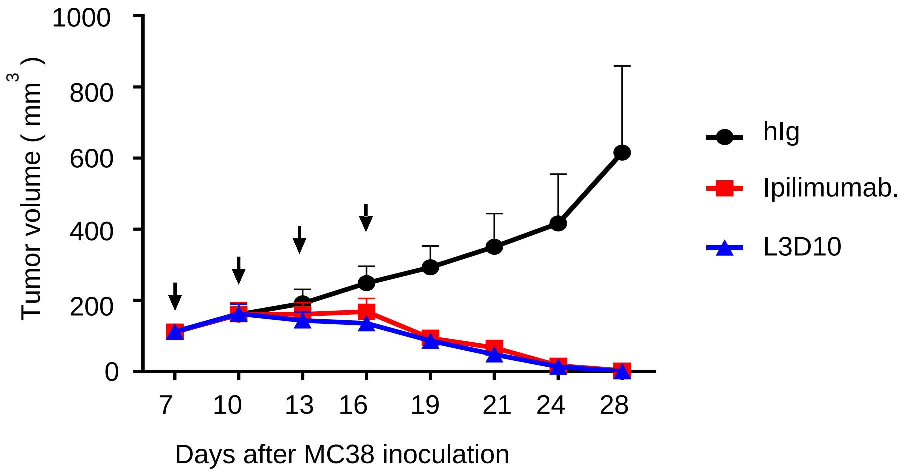

Supplementary information, Figure S1 L3D10 and Ipilimumab exhibited comparable anti-tumor activities. Tumor bearing *Ctla4*$^{h/h}$ mice (n=5) received treatment of control hIg, Ipilimumab or L3D10 (30 µg/injection × 4) on days 7, 10, 13 and 16. The tumor growth was measured every 3 days. Data are mean ± S.E.M. and have been reproduced more than 3 times. Statistical significance was analyzed by two-way repeat measurement ANOVA with Bonferroni multiple comparison test. hIg vs Ipi, P=0.0335; hIg vs L3D10, P= 0.0248; Ipi vs L3D10, P=0.6928.
