# Supplementary material for: Uncoupling therapeutic from immunotherapy-related adverse effects for safer and effective anti-CTLA-4 antibodies in CTLA4 humanized mice
Source: Cell Res. 2018 Feb 20;28(4):433–47. doi: 10.1038/s41422-018-0012-z (PMC5939041; doi:10.1038/s41422-018-0012-z)
Supplement: Supplementary file 3 — Supplementary information Figure S2 [file 41422_2018_12_MOESM3_ESM.pdf]

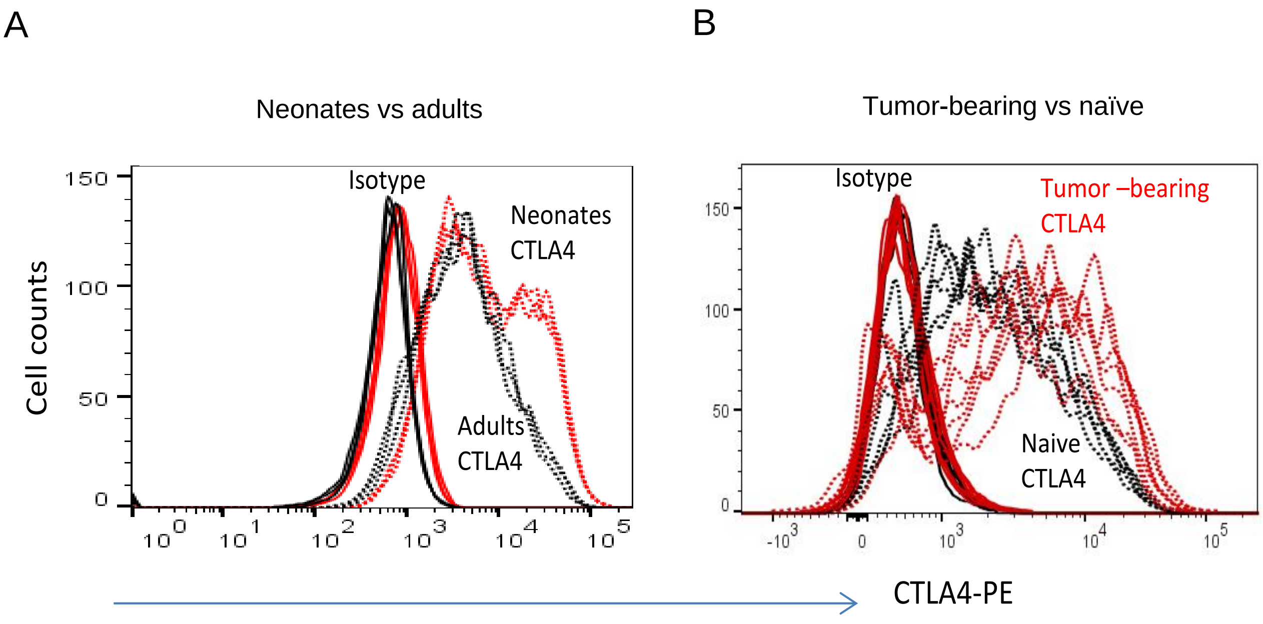

Supplementary information, Figure S2 Tregs from neonates and adult tumor-bearing mice express higher levels of CTLA-4 molecules than naïve adult mice. (**A**) Comparison between neonates (10 days old male mice, red line) and adult mice (2-3 months old male mice, black line). Data shown are profiles of Foxp3^{+}CD4^{+} Treg from spleen of male mice (n=3). (**B**) Comparison between naïve (black line) and tumor-bearing (red line) adult male mice (3 months old, n=6). Data shown are FACS profiles depicting distribution of total CTLA-4 among Foxp3^{+}CD4^{+} cells. The difference is statistically significant and has been reproduced at least five times.
