# Supplementary material for: Uncoupling therapeutic from immunotherapy-related adverse effects for safer and effective anti-CTLA-4 antibodies in CTLA4 humanized mice
Source: Cell Res. 2018 Feb 20;28(4):433–47. doi: 10.1038/s41422-018-0012-z (PMC5939041; doi:10.1038/s41422-018-0012-z)
Supplement: Supplementary file 4 — Supplementary information Figure S3 [file 41422_2018_12_MOESM4_ESM.pdf]

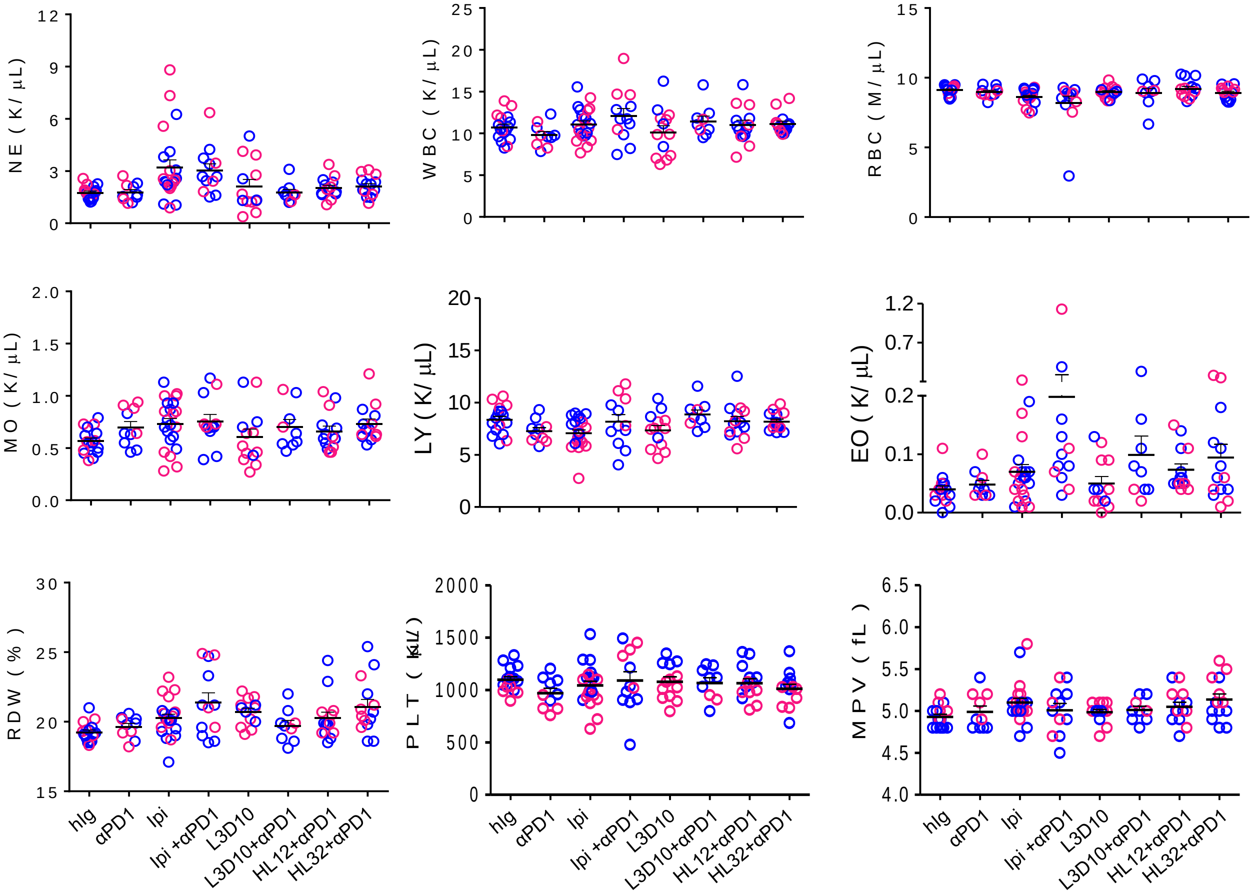

Supplementary information, Figure S3 Normal blood cell parameters following antibody treatment. Data shown are a summary of 2-3 independent experiments with each dot denotes an individual mouse (blue for male mice and red for female mice). CBC results were analyzed by Non-Parametric One-way ANOVA (Kruskal-Wallis test) with Dunn's multiple comparisons test. No statistically significant differences were found in pairwise comparisons. NE, Neutrophils; WBC, White Blood Cells; RBC, Red blood cells; MO, Monocytes; LY, Lymphocytes; EO, Eosinophils; RDW, Red cell distribution width; PLT, Platelets; MPV, Mean platelet volume.
