# Supplementary material for: Uncoupling therapeutic from immunotherapy-related adverse effects for safer and effective anti-CTLA-4 antibodies in CTLA4 humanized mice
Source: Cell Res. 2018 Feb 20;28(4):433–47. doi: 10.1038/s41422-018-0012-z (PMC5939041; doi:10.1038/s41422-018-0012-z)
Supplement: Supplementary file 5 — Supplementary information Figure S4 [file 41422_2018_12_MOESM5_ESM.pdf]

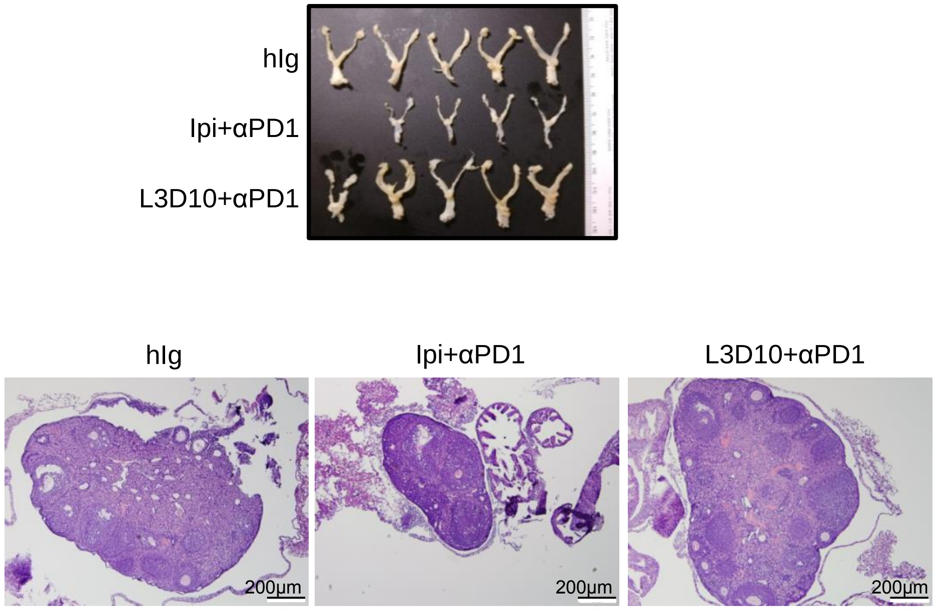


**Supplementary information, Figure S4 Gross anatomy and H&E staining show hypoplastic ovaries and uterus after Ipilimumab plus anti-PD-1 treatment**. As in Figure 1 and Figure 2, necropsy was performed on day 42 after birth.
