# Supplementary material for: Uncoupling therapeutic from immunotherapy-related adverse effects for safer and effective anti-CTLA-4 antibodies in CTLA4 humanized mice
Source: Cell Res. 2018 Feb 20;28(4):433–47. doi: 10.1038/s41422-018-0012-z (PMC5939041; doi:10.1038/s41422-018-0012-z)
Supplement: Supplementary file 6 — Supplementary information Figure S5 [file 41422_2018_12_MOESM6_ESM.pdf]

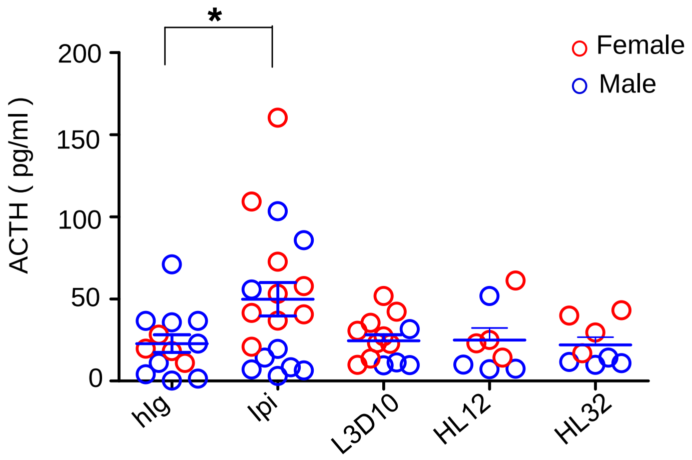

Supplementary information, Figure S5 Ipilimumab increased ACTH levels in sera. C57BL/6 *Ctla4*$^{h/h}$ mice were treated, respectively, with control human IgG Fc, anti-PD1, anti-human CTLA-4 mAbs Ipilimumab, L3D10, HL12 or HL32 at a dose of 100 μg/mouse/injection on days 10, 13, 16 and 19. Sera were collected on day 42 or 43 after birth. Serum ACTH levels were measured using Enzyme-linked Immunosorbent Assay Kit for Adrenocorticotropic Homone(Cloud-Clone Corp., Cat. No. SEA836Mu). n=8-18 mice per group. Statistical significance was analyzed by one-way ANOVA with Bonferroni multiple comparison test.
