# Supplementary material for: Uncoupling therapeutic from immunotherapy-related adverse effects for safer and effective anti-CTLA-4 antibodies in CTLA4 humanized mice
Source: Cell Res. 2018 Feb 20;28(4):433–47. doi: 10.1038/s41422-018-0012-z (PMC5939041; doi:10.1038/s41422-018-0012-z)
Supplement: Supplementary file 7 — Supplementary information Figure S6 [file 41422_2018_12_MOESM7_ESM.pdf]

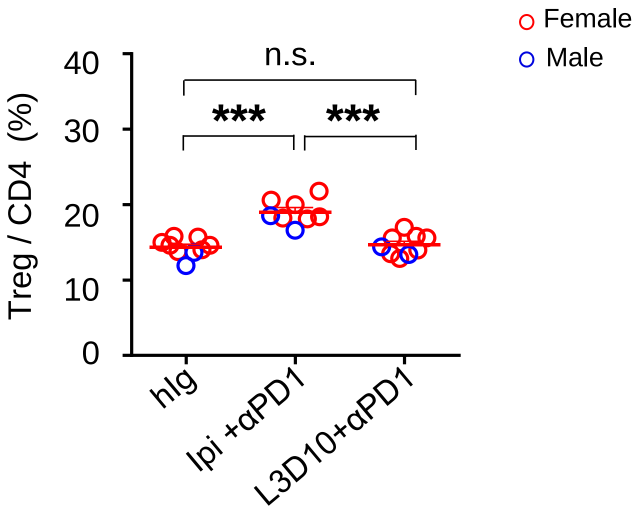


**Supplementary information, Figure S6 Ipilimumab increased the frequency of Treg in spleen from Ipilimumab-treated mice.** C57BL/6 *Ctla4*$^{h/h}$ mice were treated, respectively, with control human IgG Fc, anti-PD-1plus anti-CTLA-4 mAbs Ipilimumab or L3D10 at a dose of 100 μg/mouse/injection on days 10, 13, 16 and 19. Spleens were collected and the percentages of Foxp3$^{+}$ Treg in splenic CD4 T cells were evaluated by flow cytometry on day 42 after birth. Statistical significance was analyzed by One-way ANOVA with Bonferroni multiple comparison test.
