# Supplementary material for: Uncoupling therapeutic from immunotherapy-related adverse effects for safer and effective anti-CTLA-4 antibodies in CTLA4 humanized mice
Source: Cell Res. 2018 Feb 20;28(4):433–47. doi: 10.1038/s41422-018-0012-z (PMC5939041; doi:10.1038/s41422-018-0012-z)
Supplement: Supplementary file 8 — Supplementary information Figure S7 [file 41422_2018_12_MOESM8_ESM.pdf]

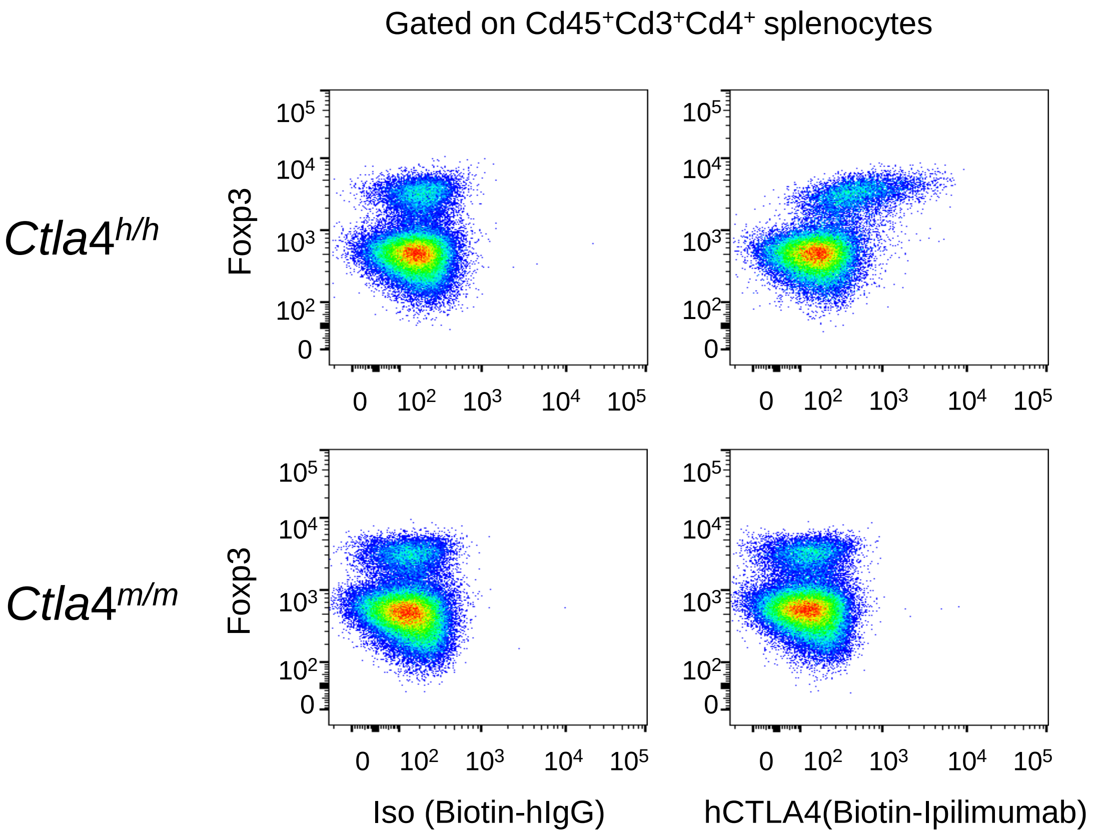

Supplementary information, Figure S7 Ipilimumab binds to human CTLA-4 but not mouse CTLA-4. Data shown are dot plots of intracellular staining of CTLA-4 among gated $CD3^+CD4^+$ cells, using spleen cells from $Ctla4^{h/h}$ (top) or $Ctla4^{m/m}$ (bottom) mice. Biotinylated hIg and Ipilimumab were used for intracellular staining. Anti-CD3 (clone 145-2C11), CD4 (clone RM4-5), FoxP3 (clone FJK-16s) mAbs and FoxP3 staining buffer were purchased from eBioscience.
