# Supplementary material for: Uncoupling therapeutic from immunotherapy-related adverse effects for safer and effective anti-CTLA-4 antibodies in CTLA4 humanized mice
Source: Cell Res. 2018 Feb 20;28(4):433–47. doi: 10.1038/s41422-018-0012-z (PMC5939041; doi:10.1038/s41422-018-0012-z)
Supplement: Supplementary file 9 — Supplementary information Figure S8 [file 41422_2018_12_MOESM9_ESM.pdf]

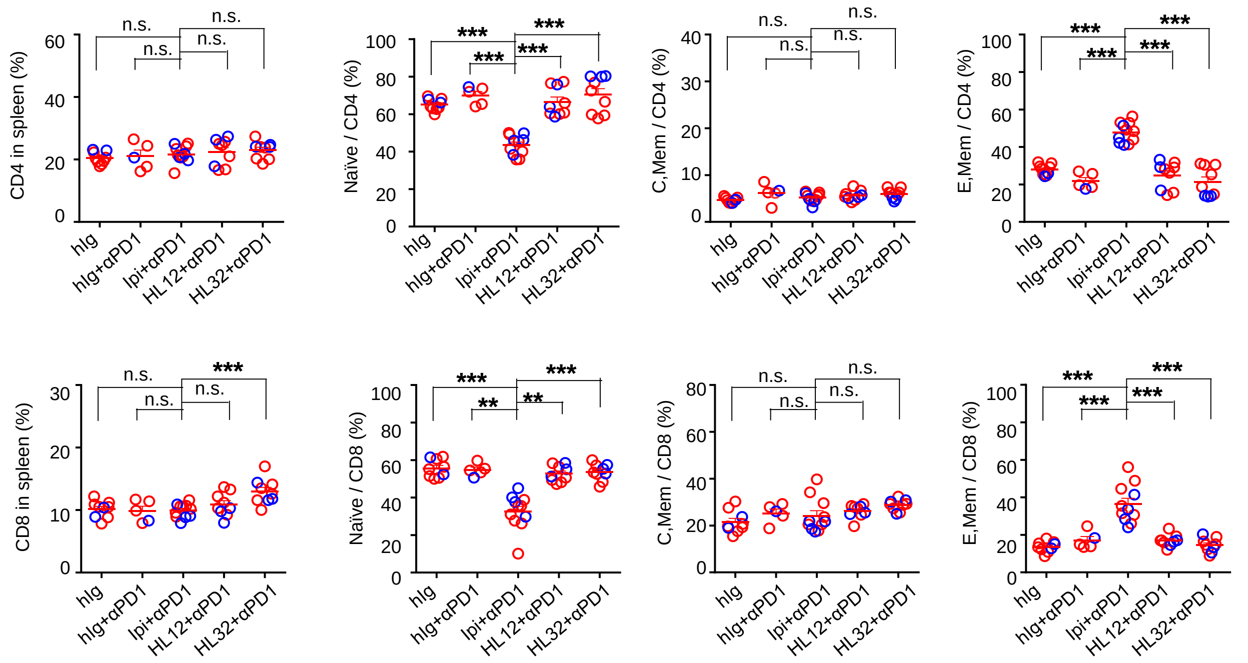

Supplementary information, Figure S8 Phenotypes of CD4 and CD8 T cells activation in the spleen of humanized mice receiving given immunotherapeutics. Mice were treated as that in Figure 1 and Figure 4, except humanized L3D10 clones (HL12 and HL32) were used. Data shown are percentages and phenotypes of CD4 (top panels) and CD8 (Bottom panels) spleen T cells on day 32 after the start of antibody treatment. Data are summarized from 3 experiments involving 5-11mice (Red: female; blue: male) per group. Statistical significance was analyzed by One-way ANOVA with Bonferroni multiple comparison test and Non-Parametric One-way ANOVA (Kruskal-Wallis test) with Dunn's multiple comparisons test.
