# Supplementary material for: Uncoupling therapeutic from immunotherapy-related adverse effects for safer and effective anti-CTLA-4 antibodies in CTLA4 humanized mice
Source: Cell Res. 2018 Feb 20;28(4):433–47. doi: 10.1038/s41422-018-0012-z (PMC5939041; doi:10.1038/s41422-018-0012-z)
Supplement: Supplementary file 10 — Supplementary information Figure S9 [file 41422_2018_12_MOESM10_ESM.pdf]

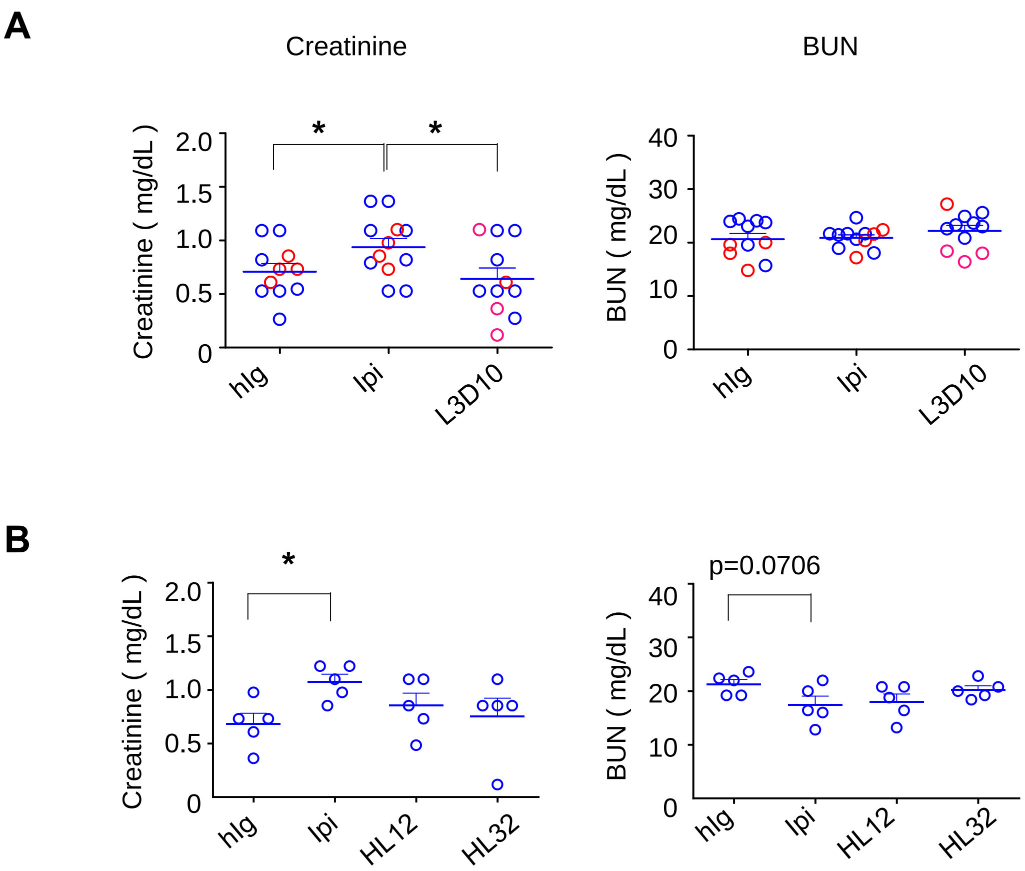

Supplementary information, Figure S9 Ipilimumab induced modest renal function abnormality in tumor-bearing mice. MC38-bearing mice were treated with 100 μg/injection/mouse for 3 or 4 times on days 7, 10, 13 and 16. Sera were collected on day 18-25 after tumor inoculation. (**A**) The levels of creatinine and BUN in sera of MC38-bearing hCTLA4-KI mice at day 18-20 (Red: female; blue: male). (**B**) The levels of creatinine and BUN in sera of MC38-bearing hCTLA4-KI mice (all male) at day 25 after tumor inoculation. Creatinine levels were measured using Creatinine (serum) Colorimetric Assay Kit (Cayman Chemical) or Creatinine (CREA) Kit (RANDOX, Cat No, CR2336). Serum BUN levels were measured using UREA NITROGEN DIRECT kit (Stanbio laboratory). Statistical significance was determined by student's *t* test.
