# Supplementary material for: Uncoupling therapeutic from immunotherapy-related adverse effects for safer and effective anti-CTLA-4 antibodies in CTLA4 humanized mice
Source: Cell Res. 2018 Feb 20;28(4):433–47. doi: 10.1038/s41422-018-0012-z (PMC5939041; doi:10.1038/s41422-018-0012-z)
Supplement: Supplementary file 11 — Supplementary information Figure S10 [file 41422_2018_12_MOESM11_ESM.pdf]

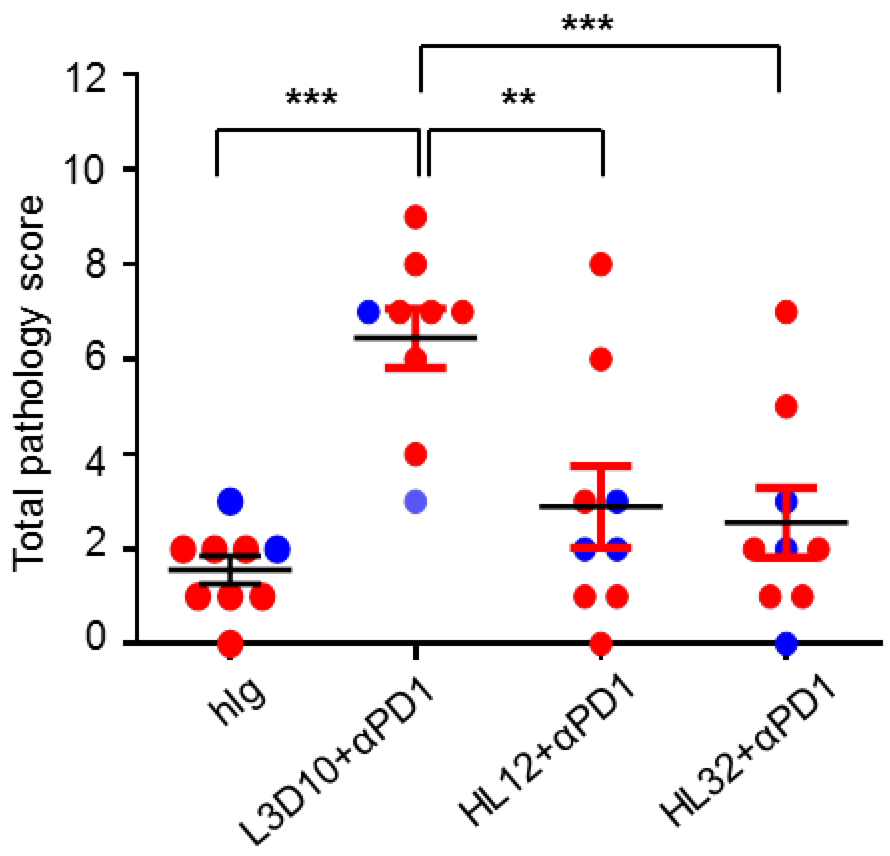

Supplementary information, Figure S10 Humanization further improves safety of L3D10 based on composite pathology scores. Blue dots represent scores of male mice and the red dots represent female mice used. All scorings were performed double blind. Data are mean ± S.E.M., and n=9 mice per group. Statistical significance was determined by one-way ANOVA with Bonferroni's multiple comparison test.
